# Translocating RNA polymerase generates R-loops at DNA double-strand breaks without any additional factors

**DOI:** 10.1093/nar/gkad689

**Published:** 2023-08-28

**Authors:** Gunhyoung Lim, Seungha Hwang, Kilwon Yu, Jin Young Kang, Changwon Kang, Sungchul Hohng

**Affiliations:** Department of Physics and Astronomy, and Institute of Applied Physics, Seoul National University, Seoul 08826, Republic of Korea; Department of Chemistry, Korea Advanced Institute of Science and Technology, Daejeon 34141, Republic of Korea; Department of Chemistry, Korea Advanced Institute of Science and Technology, Daejeon 34141, Republic of Korea; Department of Chemistry, Korea Advanced Institute of Science and Technology, Daejeon 34141, Republic of Korea; Department of Biological Sciences, and KAIST Stem Cell Center, Korea Advanced Institute of Science and Technology, Daejeon 34141, Republic of Korea; Department of Physics and Astronomy, and Institute of Applied Physics, Seoul National University, Seoul 08826, Republic of Korea

## Abstract

The R-loops forming around DNA double-strand breaks (DSBs) within actively transcribed genes play a critical role in the DSB repair process. However, the mechanisms underlying R-loop formation at DSBs remain poorly understood, with diverse proposed models involving protein factors associated with RNA polymerase (RNAP) loading, pausing/backtracking or preexisting transcript RNA invasion. In this single-molecule study using *Escherichia coli* RNAP, we discovered that transcribing RNAP alone acts as a highly effective DSB sensor, responsible for generation of R-loops upon encountering downstream DSBs, without requiring any additional factors. The R-loop formation efficiency is greatly influenced by DNA end structures, ranging here from 2.8% to 73%, and notably higher on sticky ends with 3′ or 5′ single-stranded overhangs compared to blunt ends without any overhangs. The R-loops extend unidirectionally upstream from the DSB sites and can reach the transcription start site, interfering with ongoing-round transcription. Furthermore, the extended R-loops can persist and maintain their structures, effectively preventing the efficient initiation of subsequent transcription rounds. Our results are consistent with the bubble extension model rather than the 5′-end invasion model or the middle insertion model. These discoveries provide valuable insights into the initiation of DSB repair on transcription templates across bacteria, archaea and eukaryotes.

## Graphical Abstract

**Graphical Abstract:**
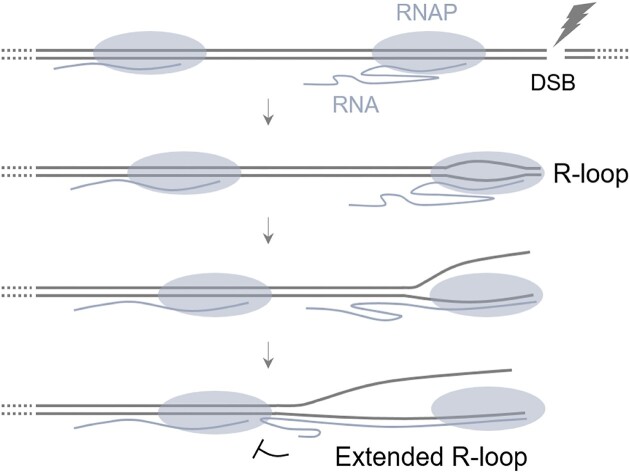

## INTRODUCTION

Cellular DNA is an exceptionally lengthy molecule that must be preserved in its entirety and faithfully replicated during each cell cycle to ensure the accurate transmission of genomic information to daughter cells. However, in each of the approximately 10^13^ cells within the human body (1,2), DNA is under constant attack from genotoxic agents originating from both internal and external sources, resulting in tens of thousands of DNA lesions per day (3,4). Among these lesions, double-strand breaks (DSBs) present a relatively small fraction, typically ranging from 10 to 50 breaks per cell a day. Nevertheless, DSBs are the most severe forms of damage and can lead to cell death if left unrepaired.

DSB repair pathways have evolved diversely, encompassing homology-directed repair (HDR), nonhomologous end joining (NHEJ), alternative end joining, single-strand annealing and microhomology-mediated template switching among others (5–7). HDR, a.k.a. homologous recombination repair, is a complex process that can result in accurate, error-free repair or gene conversion. On the other hand, NHEJ is a simpler and faster pathway but often prone to inaccuracies leading to mutations.

HDR serves the primary DSB repair pathway in bacteria and yeast (8–10). In mammalian cells, NHEJ operates throughout the cell cycle, while HDR predominantly functions during the S–G2 phases (11). However, recent studies have revealed that HDR can repair DSBs via the R-loops forming in transcriptionally active genes during the G0–G1 phases of mammalian cells (12–14), raising the question of how HDR is specifically chosen over NHEJ for DSB repair.

R-loops are three-stranded nucleic acid structures consisting of an RNA-DNA hybrid and a displaced single-stranded DNA. They were initially demonstrated *in vitro* by hybridizing a single-stranded RNA and a double-stranded DNA (15), and subsequently discovered *in vivo* in hypernegatively supercoiled plasmids isolated from an *Escherichia coli* strain with a mutation in the *topA* gene, which encodes DNA topoisomerase 1 (16,17).

R-loops pose a threat to genome integrity due to the vulnerability of the exposed single-stranded DNA regions to modifications or damages (18). Moreover, accumulation of R-loops leads to replication stress (19,20). Therefore, R-loops are considered detrimental byproducts of transcription, and their formation should be suppressed by topoisomerases and eliminated by helicases and ribonucleases (RNases).

Despite their detrimental effects on genome stability, R-loops also possess positive biological functions. R-loops have been implicated in cellular processes such as immunoglobulin class switching (21), gene regulation (22,23) and chromosome segregation (24). Furthermore, recent studies have uncovered an additional beneficial role of R-loops in transcription-coupled HDR (TC-HDR), where R-loop formation at actively transcribed genes is crucial for successful DSB repair (25–28).

To fully comprehend the involvement of R-loops in TC-HDR, it is imperative to unravel the underlying mechanisms governing R-loop formation, recognition, signaling and resolution. However, our understanding of these processes remain limited so far, and further investigation is warranted.

Multiple models have been proposed to elucidate the formation of R-loops at DSBs (29). One model known as the DSB as promoter model suggests that RNA-DNA hybrids are generated through the de novo bidirectional transcription initiated at DSB ends acting as unconventional promoters. This model proposes that binding of MRE11-RAD50-NBS1 complexes to DSB ends facilitates recruitment of RNA polymerase (RNAP) II and III, promoting transcription in the vicinity of DSBs (26,30).

Another model called the transcription regulation model suggests that R-loops form as a result of repression of ongoing transcription near DSBs. It has been observed that DSBs can trigger transcription repression (31,32), and this model proposes that the reduced elongation rate and increased pausing contribute to R-loop formation (28).

In addition to these models, two other hypotheses have been proposed. The RNAP II backtracking model suggests that transcriptional backtracking occurs due to obstacles on DNA (33), leading to R-loop formation. The strand invasion model suggests that Rad51 homologues mediate the invasion of preexisting transcript RNA into double-stranded DNA, generating R-loops (27,34).

Despite the presence of circumstantial evidence supporting these models, the exact mechanism of R-loop formation at DSBs has remained elusive. This is primarily due to the lack of comprehensive biochemical studies that directly demonstrate R-loop formation at DSBs. Further research with systematic *in vitro* experiments is required to gain a deeper understanding of the precise mechanisms underlying R-loop formation at DSBs.

This study directly examines how R-loops form at DSB ends and our results resolve several current controversies. We discover that *E. coli* RNAP alone possesses the ability to sense DSBs and generate R-loops during transcription. We additionally demonstrate upward extension of R-loops and RNA-DNA hybrids from DSB sites. Furthermore, we show that such R-loops interfere with both ongoing and subsequent rounds of transcription. Thus, our findings shed light on a previously unknown capability of translocating RNAP to detect DSBs on transcription templates for DNA repair.

## MATERIALS AND METHODS

### Preparation of DNA templates for transcription

To construct the transcription templates used in the study, the template and nontemplate strands were generated by ligating two or three specific oligonucleotides. All oligomers were obtained from Integrated DNA Technologies and their sequences can be found in Supplementary Table S1. The process of ligating the oligomers involved annealing them to a splint DNA in a buffer containing 10 mM Tris–HCl, pH 8.0 and 50 mM NaCl. The annealing was performed by slowly cooling the mixture from 95 to 4°C. Subsequently, the phage T4 DNA ligase from New England Biolabs was used to catalyze the ligation reaction at 16°C for 16 h.

The ligation products were purified using denaturing gels, and the resulting two strands were annealed together in a buffer consisting of 20 mM Tris–HCl, pH 8.0 and 20 mM LiCl. The annealed strands were then stored at −20°C until further use. The 1161-bp DT8 template was constructed by ligating a 1002-bp DraIII fragment of the phage λ DNA to the DT3 template at the downstream end. The ligation product was subsequently amplified by polymerase chain reaction.

### Preparation of fluorescent stalled transcription complexes

*E. coli* RNAP core enzyme and RpoD, a.k.a. σ^70^, were obtained through the custom purification methods described previously (35). Alternatively, the RNAP holoenzyme was purchased from New England Biolabs. To prepare the stalled elongation complexes, a mixture containing 340 nM RNAP holoenzyme, 50 nM DNA template, 100 μM Cy3-ApU, 60 μM CTP and 60 μM UTP was incubated in a buffer composed of 40 mM Tris–HCl, pH 8.0, 50 mM KCl, 20 mM MgCl_2_ and 10 mM dithiothreitol at 37°C for 40 min.

### Single-molecule fluorescence transcription experiments

The microscope quartz slides and glass coverslips were cleaned using piranha solution, a mixture of concentrated sulfuric acid and hydrogen peroxide. They were then silanized with 3-aminopropyltrimethoxysilane and coated with a mixture of PEG-5000 and biotin-PEG-5000 in a ratio of 40:1 (Laysan Bio). A microfluidic detection chamber with a volume of 20 μl was assembled with the cleaned slide and coverslip. A sterilized polyethylene tubing was connected to the inlet and outlet of the chamber to enable fast buffer changes at a flow rate of 2 ml/min using a syringe pump (Fusion 100, Chemyx Inc.) and the exchange took 0.6 s.

The stalled complexes were injected into the microfluidic chamber and immobilized on the surfaces via biotin-streptavidin conjugation. After removing the unbound complexes by washing, real-time single-molecule fluorescence images were acquired using a home-made total-internal-reflection fluorescence microscope. The imaging buffer contained 40 mM Tris–HCl, pH 8.0, 50 mM KCl, 5 mM NaOH, 20 mM MgCl_2_, 1 mM dithiothreitol, 2 mM spermidine, 3 mM trolox, 5 mM protocatechuic acid and 4 units/ml protocatechuate-3,4-dioxygenase (Oriental Yeast Co.). To resume transcription and monitor R-loop formation, the imaging buffer was supplemented with 2 mM NTPs, monoclonal antibody S9.6 and Alexa Fluor 488-labeled secondary antibody. The buffer solution was injected into the flow cell after incubation at 37°C for 3 min.

Fluorescence excitation of Alexa-488, Cy3 and Cy5 was achieved using solid-state lasers emitting at wavelengths of 473, 532 and 640 nm, respectively. The fluorescence signals were recorded using a camera (iXon DV897ECS-BV from Andor Technology) with a binning time of 0.2 or 0.5 s. The temperature of the prism, detection chamber, objective lens and injected buffer solution was maintained at 37°C using a control system from Live Cell Instrument.

Data analysis was performed using custom programs written in IDL 7.0 (ITT) or MATLAB R2013b (The MathWorks), as well as commercial software such as Origin 8.0 (OriginLab) and Sigma Plot 8.0 (Systat Software). The numbers of analyzed molecules and replicates in each experiment are provided in Supplementary Tables S2 to S4.

## RESULTS

### Single-molecule monitoring of R-loop formation

**Figure 1.**
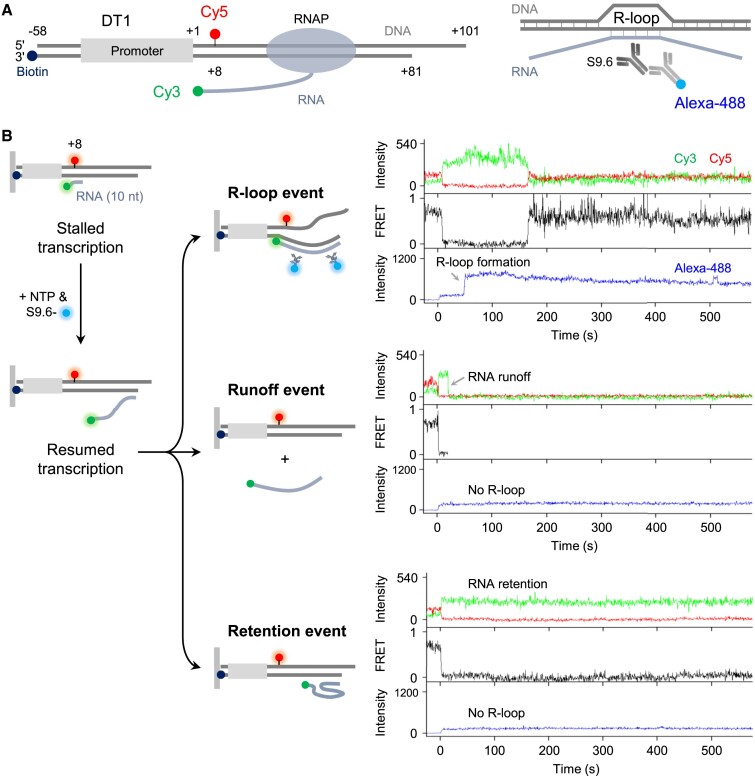
Single-molecule observation of cotranscriptional R-loop formation. (**A**) Fluorescent detection of active transcription and R-loop formation. Double-fluorescent transcription complexes each contain *E. coli* RNAP holoenzyme, a Cy3-labeled RNA transcript and a Cy5-labeled DNA template with the T7A1 promoter. The specific changes in Cy3 and Cy5 fluorescence indicate the presence of active transcription complexes. Additionally, the monoclonal antibody S9.6, which specifically binds RNA-DNA hybrids, is bound to an Alexa-488-labeled secondary antibody. This primary/secondary antibody complex allows for the specific detection of R-loops. (**B**) Experimental scheme and three outcomes. Stalled complexes, formed by incubating a DNA template with RNAP holoenzyme, Cy3-ApU, CTP and UTP, are immobilized on a microscope slide. After the injection of NTPs to resume transcription, R-loop formation is monitored for 10 min using the Alexa-488-labeled antibody. In addition to the R-loop formation that accompanies Alexa-488 signal appearance (R-loop events), transcript RNA dissociation from DNA (runoff events) and occasionally prolonged RNA retention on DNA without R-loop formation (retention events) are observed. For each event, shown on the right are the representative time traces of Cy3 and Cy5 fluorescence at Cy3 excitation (top), Cy3–Cy5 FRET (middle) and Alexa-488 fluorescence at its excitation (bottom) since the NTP injection at 0 s.

In order to observe the formation of R-loops during transcription at the single-molecule level in real time, we prepared several specific linear DNA templates, referred to as DT1 through DT8, for our fluorescence experiments. To start with, DT1 contains the T7A1 promoter at upstream of the transcription start site +1 and a G-quadraplex forming sequence at positions +13 to +27 but lacks any terminator sequence (Figure 1A, left).

The downstream end of DT1 contains a sticky region with a 20-nt 3′-overhang at positions +81 to +101, mimicking the broken ends of most natural DSBs. The upstream end at position −58 is labeled with biotin to enable surface immobilization, while the nontemplate strand base at position +8 is labeled with Cy5 for single-molecule monitoring. These modifications and labeling enable us to study both the movement of RNAP and the formation of R-loops during transcription on this specific DNA template.

In our fluorescence experiments, we incubate the Cy5-labeled DNA template with Cy3-labeled ApU dinucleotide, CTP, UTP and *E. coli* RNAP holoenzyme containing σ^70^. When transcription initiates with Cy3-ApU, the product transcript RNA carries a Cy3 label at its 5′-end. Due to the absence of ATP and GTP, the transcription process stalls with temporary production of a 10-nt RNA transcript (36,37). The stalled complexes are immobilized on a microscope slide via biotin-streptavidin conjugation and subsequently washed before we begin to capture real-time fluorescence images.

In the double-fluorescent stalled complexes, the Cy3-labeled RNA 5′-end is in close proximity to the Cy5-labeled nontemplate DNA base at position +8. This spatial arrangement enables fluorescence resonance energy transfer (FRET) to occur efficiently from Cy3 (donor) to Cy5 (acceptor), resulting in a measurable FRET signal with an average efficiency (*E*_FRET_) of 0.69 (Supplementary Figure S1).

The stalled transcription is resumed by injection of all four NTPs, which is the zero time point in the experiments. Upon transcription resumption, the FRET signal abruptly decreases because the Cy3-labeled RNA 5′-end moves away from the Cy5-labeled DNA +8-base. This stepwise decline in FRET was observed in 97% of the FRET complexes (Supplementary Figure S1), indicating their active participation in transcription. These active complexes were exclusively analyzed in the study.

In order to detect the formation of R-loops, we utilized the RNA-DNA hybrid-specific monoclonal antibody S9.6, which was fluorescently stained with an Alexa Fluor 488-labeled secondary antibody (Figure 1A, right). The antibody binding to RNA-DNA hybrids is rapid, taking only 6.6 s (Supplementary Figure S2A). It is specific, as nonspecific binding to slide surfaces is negligible (Supplementary Figure S2B). Therefore, the Alexa-488 fluorescence signal reliably reports the R-loop formation in real time.

We observed three different outcomes during 10-min monitoring (Figure 1B). First, R-loop forms with transcript RNA. This R-loop event is characterized by Alexa-488 signal appearance. Second, RNA dissociates from the immobilized DNA, leading to runoff transcription. This runoff event is identified by Cy3 signal loss without Alexa-488 signal. Third, RNA retains on DNA without forming an R-loop. This retention event is classified by Cy3 signal maintenance without Alexa-488 signal. Thus, each and every active complex is counted as one of the three events.

Using the DT1 template, we observed R-loop events in 73% of the active complexes (Figure 1B and Supplementary Figure S3), which we refer to as R-loop efficiency. Most R-loops form in low-FRET states (Supplementary Figure S4A), denoting that RNA regions other than the 5′-end can join R-loops. In some complexes, FRET rises again some time after Alexa-488 signal appears (Figure 1B and Supplementary Figure S3), indicating that some R-loops include the RNA 5′-end as well. The 5′-end inclusion could be stringently assured by *E*_FRET_ > 0.7, and such a high-FRET population gradually increases over time implying that more and more complexes have an R-loop with the 5′ end, although it does not become dominant (Supplementary Figure S4B).

The S9.6 antibody binding starts at 74 s since the NTP injection (Supplementary Figure S2C), referring to the interval between transcription resumption and R-loop formation. In the R-loop events with a high FRET, the first antibody binding is followed by additional bindings (Supplementary Figure S2D), suggesting that this population has large extended R-loops that possibly contain both 5′ and 3′ ends of transcript RNA.

When supplemented with RNase H, which specifically digests the RNA strand hybridized to a DNA strand, the high-FRET population readily disappears (Supplementary Figure S5), confirming that the high-FRET state that we observed corresponds to R-loop structure. The high-FRET population is hardly affected by the presence of the primary/secondary antibody complex (Supplementary Figure S6), attesting that the antibody binding does not actively assist but just passively detects R-loop formation.

In the runoff events, R-loops do not form and Cy3-labeled transcripts rapidly dissociate from DNA after RNAP reaches the downstream end (Figure 1B and Supplementary Figure S3). Cy3 signal disappears at 15 s since the NTP injection (Supplementary Figure S7). This signal duration, here called runoff time, is the sum of the transcription elongation time taken from the stalling site to the downstream end plus the RNA dissociation time taken at the downstream end. In short DNA templates with a negligible elongation time, the runoff time practically represents the RNA dissociation time.

Lastly, the retention events, which exhibit prolonged retention of RNA on DNA for the entire 10-min duration, are infrequent on the DT1 template (Figure 1B and Supplementary Figure S3). This fraction could be converted in principle to either the R-loop event or the runoff event at any time after the 10-min monitoring, unless remains unchanged. However, the investigation of the underlying mechanism for this occasionally extended RNA retention is beyond the scope of this study.

### R-loop formation at DSB sites by translocating RNAP

**Figure 2.**
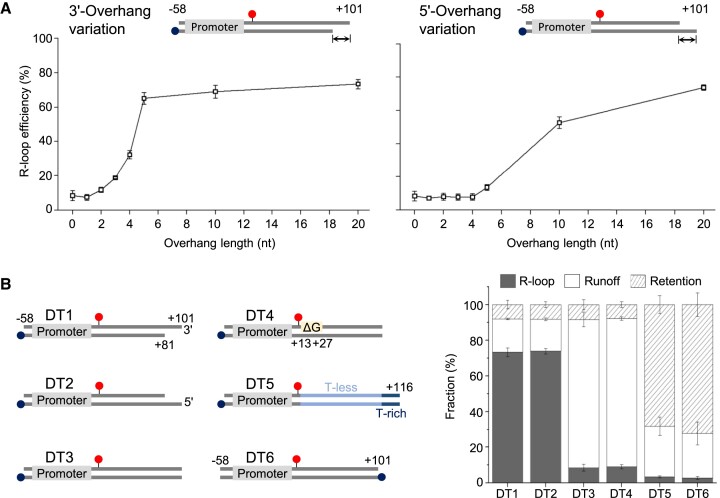
DSB sensing and R-loop generation by RNAP. (**A**) The overhang length dependence of R-loop formation efficiency. The R-loop efficiencies on the y-axis are plotted against the overhang lengths on the x-axis and shown separately for templates with a 3′-overhang (left) and those with a 5′-overhang (right). (**B**) R-loop efficiencies of various DNA templates. Fractions of the R-loop, runoff and retention events on each template are shown. DT1 and DT2 have a 20-nt 3′ or 5′-overhang, respectively at the downstream end. DT3 has a blunt downstream end. In DT4, a G-rich sequence is deleted (ΔG). DT5 has an inserted T-less sequence. DT6 has biotin attached to the downstream end instead of the upstream end. The sequences of all the templates can be found in Supplementary Table S1.

Using this newly established single-molecule assay, we aimed to investigate the factors contributing to efficient R-loop formation. We initially examined two sets of templates with varying lengths of 3′- or 5′-overhangs, ranging from 20 to 1 nt (Figure 2A), while the template with a 20-nt 5′-overhang downstream end is named DT2. Regardless of whether the overhangs are at the 3′- or 5′-end, R-loop efficiency decreases as the overhang length shortens. Even with a blunt-end template DT3 without any overhang, R-loop still forms, albeit with a reduced efficiency of 8.4%.

In order to identify the source(s) of the residual R-loop efficiency exhibited by the blunt-ended DNA, we generated three variants DT4 to DT6 by modifying DT3 (Figure 2B). In DT4, the G-quadruplex forming sequence is deleted by replacement with a non-forming sequence. The R-loop efficiency shows little difference between DT3 and DT4, suggesting that the G-rich sequence does not much contribute to R-loop formation on DT3.

DT5 contains an inserted T-less sequence, causing transcription elongation to stall just beyond it in the absence of UTP. DT6 has the biotin located at the downstream end instead of the upstream end, leading to stalling at the conjugated streptavidin. Both DT5 and DT6 exhibit increased transcription stalling and prolonged RNA retention compared to DT3 (Supplementary Figure S7), as expected. However, the R-loop efficiency decreases, indicating that R-loop formation is not significantly induced by stalling or prolonged pausing but is instead facilitated by the presence of a free broken end.

Therefore, while R-loops can form at the DSB sites with blunt ends to a certain degree or a basal level, they are much more efficiently generated at sticky ends with any single-stranded overhangs of sufficient lengths. Apparently, the longer DSB end overhangs are, the more efficiently R-loops form. Most importantly, high efficiencies of R-loop formation can be achieved by transcribing RNAP alone without assistance from any other factors.

### Upward expansion of R-loops from DSB sites

Several mechanisms can be envisaged for cotranscriptional R-loop formation, differing in the origin and direction of expansion; extension of transcription bubble, reannealing by 5′-end invasion and reannealing in the middle (38). Among these models, the 5′-end invasion model is deemed unlikely to be a major mechanism since R-loops predominantly form at low-FRET states across various DNA templates (Supplementary Figures S4A and S8).

**Figure 3.**
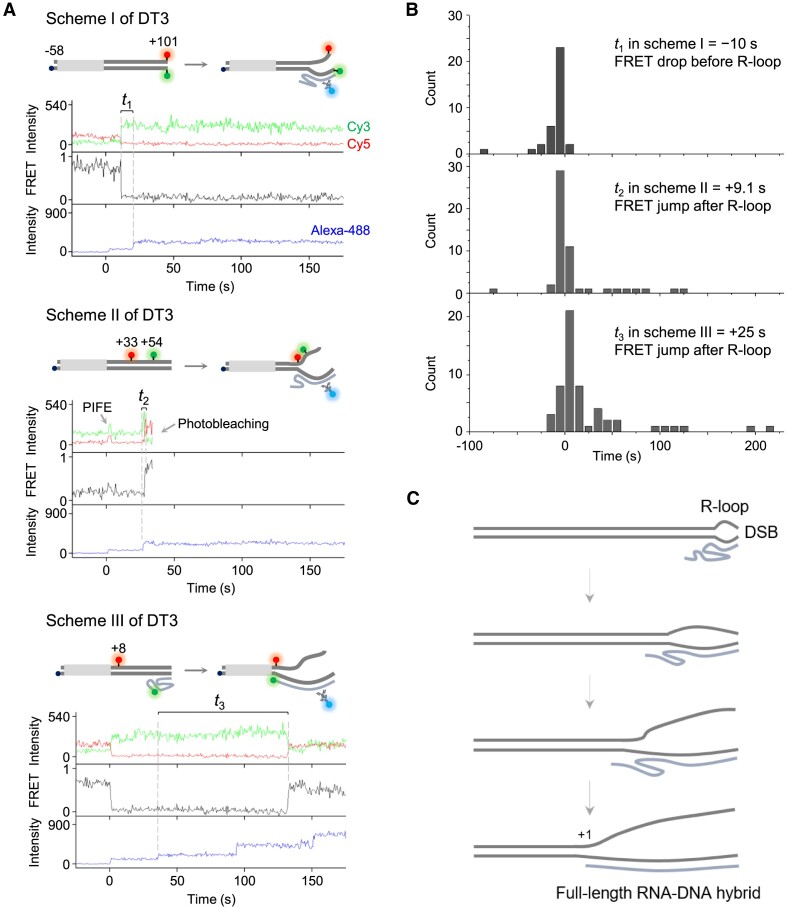
Extension of RNA-DNA hybrids from DSBs. (**A**) Three different schemes of fluorophore labeling. Representative time traces of Cy3 and Cy5 fluorescence at Cy3 excitation (top), Cy3-Cy5 FRET (middle) and Alexa-488 fluorescence at its excitation (bottom) are shown for each scheme for DT3. In scheme II, transient PIFE occurs due to the encounter of transcribing RNAP with Cy3 and Cy5, and time traces of Cy3 and Cy5 fluorescence and corresponding FRET are truncated after their photobleaching. (**B**) Timing of FRET changes with respect to antibody detection of R-loops in the schemes I (top), II (middle) and III (bottom). The average values are presented in the figure. (**C**) Our model of R-loop formation and extension. R-loop formation starts with the 3′-end of transcript RNA and the downstream end of template DNA, representing a DSB site. The R-loop extends upward from the DSB site as the upstream border progressively migrates towards the 5′-end of RNA while the downstream border remains with the 3′ end of RNA.

In order to further explore the remaining models, we labeled the DT3 template with Cy3 and Cy5 at varying positions to monitor R-loop formation at the downstream end, a middle region and a further upstream position separately (Figure 3A and Supplementary Figure S9). The Cy3→Cy5 FRET alters depending on the positions of the labeled dyes and the movement of the R-loop.

In scheme I, both Cy3 and Cy5 dyes are placed at the downstream end but on separate strands of DNA, allowing for FRET to occur efficiently. When an R-loop forms at the downstream end and separate the two strands there, the FRET signal drops at 10 s before the R-loop is detected by the S9.6 antibody (Figure 3B).

In scheme II, both dyes are labeled on the DNA nontemplate strand, specifically at the +54 and +33 positions separately. When the R-loop covers this middle region, *E*_FRET_ increases from 0.21 to 0.64 (Supplementary Figure S10). The FRET signal rise in this scheme occurs at 9.1 s after the antibody binding.

Scheme III is the same as Figure 1A with Cy3 at the RNA +1 position and Cy5 at the DNA +8 position. When the R-loop reaches the +1 site, the RNA 5′-end approaches the +8-base and FRET ascends to *E*_FRET_ of >0.7, which is detected at 25 s after the antibody binding. Additionally, with the DT1 and DT2 templates in scheme III, the FRET signal arises similarly at 105 and 31 s, respectively after the antibody binding (Supplementary Figure S11).

Using the timings of the three local R-loop detections measured with the DT3 template, the R-loop extension speed appears to be a few bp/s within an order of magnitude. In essence, the FRET changes are first detected at the +101 position before the antibody detects the R-loop, and subsequently observed at the +33 and +1 positions sequentially following the first detection. Therefore, the R-loop grows from the downstream end towards upstream sites, and eventually, the transcript's 5′-end joins the R-loop at the upstream edge (Figure 3C).

Similar results were obtained when we repeated the experiments using another template named DT7 with a 5-nt 3′-overhang downstream end (Supplementary Figure S12). All these findings support the bubble extension model, a.k.a. extended hybrid model, where R-loops and RNA-DNA hybrids extend upstream from DSB sites, and the expansion can eventually reach the +1 site.

### Interference of R-loops with transcription process

**Figure 4.**
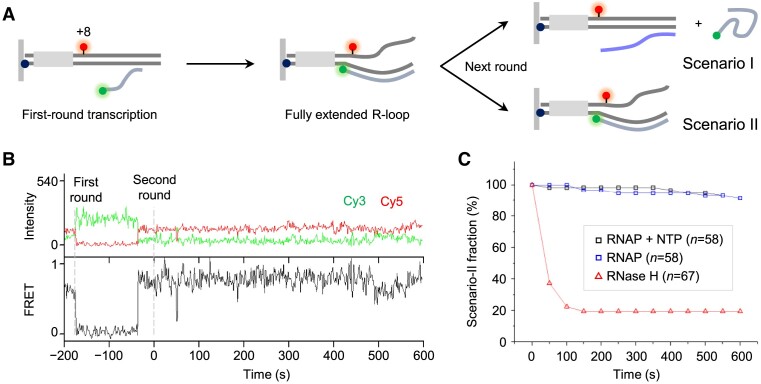
Blocking effect of R-loops on the next-round transcription. (**A**) Experimental setup with DT3. RNAP holoenzyme and NTPs are injected for multiple-round transcription. (**B)** Representative time traces of Cy3 and Cy5 fluorescence at Cy3 excitation (top) and Cy3-Cy5 FRET (bottom). The traces depict the fluorescence signals during multiple-round transcription after the injection of RNAP holoenzyme and NTPs at 0 s. (**C**) R-loop maintenance of the scenario II. Most R-loops retain the Cy3 signal even when the next-round transcription is permitted, whereas they rapidly disappear when RNase H is added. This indicates that R-loops can persist and maintain their structures, effectively preventing the initiation of subsequent transcription rounds.

In light of a recent paper alluding that R-loops enhance transcription (39) contrary to other previous studies (16,40,41), we aimed to investigate the controversial effects of R-loops on transcription process (Figure 4A). The DT3 template complexes with fully expanded R-loops exhibit high Cy3→Cy5 FRET, indicating the proximity of the Cy3-labeled RNA 5′-end to the Cy5-labeled +8-base (Figure 4B and Supplementary Figure S13). After a 3-min single-round transcription of DT3, we added RNAP holoenzyme and NTPs without Cy3-ApU for subsequent rounds of transcription to proceed. As a control, we added the holoenzyme alone without NTPs.

Two scenarios can be considered. In scenario I, if the added RNAP starts the second round from the promoter, the RNA-DNA hybrid unwinds and the Cy3-labeled transcript diffuses away, resulting in disappearance of the Cy3 signal. In scenario II, if the second-round transcription is inhibited by the extended R-loop, the Cy3 signal would persist.

Among the complexes exhibiting FRET, the population retaining the Cy3 signal is minimally reduced over 10 min and shows little difference from the control (Figure 4C). Moreover, it readily disappears after RNase H treatment, validating the persistence of R-loop (Figure 4C). These results assert that extended R-loops effectively block subsequent rounds of transcription.

**Figure 5.**
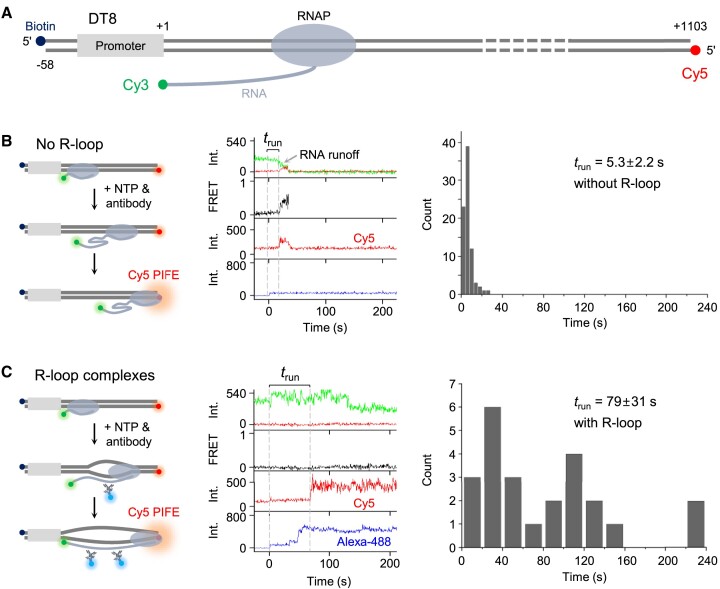
Hindrance of the ongoing-round transcription by R-loops. (**A**) A long DNA template DT8. The downstream end is labeled with Cy5 and the transcript 5′-end with Cy3. (**B**) Ongoing-round transcription in the absence of R-loop formation. Shown are a schematic diagram (left), a representative time trace (center) and the measurement of RNAP running time (*t*_run_) to the downstream end (right). The time trace displays Cy3 and Cy5 fluorescence at Cy3 excitation (top), Cy3-Cy5 FRET (second), Cy5 fluorescence at its excitation (third) and Alexa-488 fluorescence at its excitation (bottom) since the NTP injection at 0 s. *t*_run_ is measured from the NTP injection to the start of Cy5 PIFE. The average value is presented in the figure. (**C**) Hindrance of ongoing-round transcription in the presence of internal R-loop formation. Shown are a schematic diagram (left), a representative time trace (center) and the measurement of *t*_run_ (right). Similar to (B), the time trace shows the fluorescence signals of Cy3, Cy5 and Alexa-488. However, in this case, the internal R-loops form. The delay in Cy5 PIFE starting indicates that the ongoing-round transcription is significantly hindered by the presence of internal R-loops.

Next, we examined the effect of R-loops on the ongoing round of transcription, which has not been extensively studied so far. R-loops can form not only at the broken ends (terminal R-loops) but also in the middle of templates, particularly in the regions prone to R-loop formation (internal R-loops). Thus, R-loops can form more abundantly in longer templates than shorter ones. We prepared a long DNA template called DT8 where the downstream end at position +1103 is labeled with Cy5 and the upstream end at position −58 with biotin, and the product transcripts got labeled with Cy3 at the 5′-end (Figure 5A).

Cy5 displays protein-induced fluorescence enhancement (PIFE) when its *trans*→*cis* photoisomerization is restricted due to the approach of translocating RNAP to the downstream end of this terminator-less template (42,43). The R-loop forming complexes with Alexa-488 signal comprise 18% of the total complexes with both Cy3-RNA and Cy5-DNA. In the remaining 82% complexes, where no R-loop forms, Cy5 PIFE starts at 5.3 s, which is taken for transcribing RNAP to translocate to the downstream end from the stalling site (Figure 5B and Supplementary Figure S14).

In 11% of the R-loop forming complexes, the Alexa-488 signal appearance clearly precedes the Cy5 PIFE starting, which reports the internal R-loop formation prior to RNAP’s arrival at the downstream end. With these internal R-loops, the Cy5 PIFE starting is delayed by a factor of 15, occurring at 79 s (Figure 5C and Supplementary Figure S14). The internal R-loops form much more likely upstream than downstream of the translocating RNAP. Thus, this delay indicates that ongoing-round transcription is substantially impeded by the internal R-loops trailing behind the elongating RNAP.

In conclusion, the R-loops that form during transcription appear to interfere with both current and subsequent rounds of transcription. The terminal R-loops forming at the broken ends can expand upward to enclose the +1 site, preempting next-round transcription of the damaged templates, while the internal R-loops forming in the middle of templates can slow down the downstream RNAP at work. That is to say, the R-loops placed anywhere on template DNA molecules obstruct rather than permit or facilitate transcription process.

## DISCUSSION

While the recognition of DNA damages is the crucial first step in any DNA repair process, here we present the evidence that transcribing RNAP alone serves as an efficient sensor for the DSBs of transcription template DNA. RNAP effectively detects the lesions in expressed genes and operons as it scans them during transcription. Consequently, RNAP generates R-loops and expands them to large sizes, even encompassing the full-length transcripts. Functionally, such R-loops can stop transcription of the damaged DNA templates and could call for a pertinent repair process.

Remarkably, RNAP plays an active role in both transcription-coupled nucleotide excision repair (TC-NER) of single-strand damages and TC-HDR of double-strand damages. During TC-HDR, RNAP engages in the formation of R-loops containing extended RNA-DNA hybrids, whereas in TC-NER, RNAP merely stalls. As the stalling RNAP recruits NER factors, the R-loops or RNAPs bound to them may interact with HDR factors, impeding the current and subsequent transcription rounds.

R-loops have been regarded as a threat to genome stability but recent discoveries have positioned them as critical intermediates in TC-HDR of DSBs (25–28). Nevertheless, mechanistic questions surrounding R-loop formation, recognition, signaling and resolution during TC-HDR have remained largely unknown. This single-molecule study settles various existing disputes by directly testing how R-loops form at DSBs in a minimal setting only with the basal components of cellular transcription machinery, without any auxiliary factors for transcription or repair.

We have observed here that the efficiency of R-loop formation greatly depends on the DNA end structures, ranging from 2.8% to 73%, and tends to be inversely correlated with RNA runoff efficiency, as demonstrated with the DT1∼DT6 templates in Figure 2B. R-loop formation is dominant on the sticky ends with either 3′- or 5′-single-stranded overhangs of sufficient lengths, while RNA runoff prevails from the blunt ends without any overhangs or the sticky ends with short overhangs. These results highlight for the first time how single-stranded overhangs at DSBs play a major role in R-loop formation, aligning well with the prevalence of sticky ends as natural DSB ends rather than blunt ends.

Among the several previously proposed pathways of cotranscriptional R-loop formation (38), our experimental results align more closely with the bubble extension model, a.k.a. extended hybrid model, than the 5′-end invasion model and the middle insertion model. Specifically, the R-loop bubbles and RNA-DNA hybrids extend in size with their upstream border moving unidirectionally upward from the melted downstream end (Figure 3C).

Once transcribing RNAP arrives at the template's downstream end, which serves as a DSB end, a small RNA-DNA hybrid can form, possibly via the upward expansion of the normal transcription bubble in the elongation complex. Subsequently, the R-loop expands as upstream regions progressively join the RNA-DNA hybrid, ultimately completing the R-loop expansion when the transcript 5′-end gets enclosed finally. This unidirectional upward expansion of R-loops from DSB sites, as depicted in Figure 3C, would represent a major step in the cellular TC-HDR pathway.

Structurally, in the elongation bubble, the two strands of RNA-DNA hybrid get separated in the upstream tail by the lid loop domain of RNAP. This separation assists reannealing of the upstream DNA and is commonly observed in *E. coli*, human and other transcription complexes (44–46). While further structural studies are anticipated, we hypothesize that when translocating RNAP reaches a broken end of DNA, its structure undergoes the changes that disable the strand separation of RNA-DNA hybrid, potentially due to the lack of energy compensation from the upstream DNA rewinding. Consequently, the hybrid in the elongation bubble becomes readily extendable in the upstream direction.

This hypothesis finds support in our observations of the gradual upward extension of R-loops in this study, as well as the previous identification of R-loop formation during transcription of single-stranded templates using an RNAP variant lacking the lid loop (47). The failure of strand separation at the upstream edge of the RNA-DNA hybrid likely contributes to R-loop formation, and the lid loop, responsible for hybrid strand separation, exhibits structural conservation from bacteria to archaea and eukaryotes (48,49). Thereupon, our findings with *E. coli* RNAP in this study could be applicable to human and other RNAPs in terms of their ability to sense DSBs and generate R-loops for TC-HDR.

Cotranscriptional R-loop extension has been presumed to face topological hindrance as DNA template strand switches its pairing partner from DNA nontemplate strand to nascent RNA. While the transcribing RNAP interacts with both template and nontemplate strands, RNA should pass through the gap between the two DNA strands for R-loop extension (50). However, in our model of R-loop formation and extension (Figure 3C), the template strand is separated from the nontemplate strand around the downstream end so could readily wind around RNA in R-loop extension without much topological burden.

Recently, there has been a debate regarding the influence of R-loops on subsequent rounds of transcription, as a study reported transcription stimulation by R-loop (39). However, we here demonstrate that extended R-loops completely block the next transcription rounds. Furthermore, we observed here for the first time that among the internal R-loops, the upstream trailing R-loops can somehow interfere with the ongoing transcription round, while the hindrance by the downstream leading R-loops has been previously envisioned (51). We speculate that this suppression of inappropriate transcription across DSBs helps conserve cellular resources for DNA repair.

Our single-molecule fluorescence assays developed here can be used to address various questions related to R-loop formation, extension, recognition, signaling, disassembly and degradation among others. First, R-loop formation and extension would generally require superior thermodynamic stability of RNA-DNA heteroduplexes over DNA-DNA homoduplexes, which notably depends upon DNA/RNA sequences (52,53). For example, certain G-rich sequences reportedly enhance R-loop efficiency (54). Our assays can directly and quantitatively examine such sequence dependence of R-loop efficiency.

Second, R-loops or RNA-DNA hybrids could play an essential role of recruiting the relevant repair system to DSB sites and enabling the cellular TC-HDR pathway. Our single-molecule assays can study the interactions of R-loops or RNAPs bound to them with the repair system for the R-loop recognition and signaling steps.

Third, R-loop would be a transient or transitory structure in live cells. After fulfilling the recruiting role, R-loop structures would be resolved eventually through either disassembly by helicases, degradation by nucleases or both. Our assays can study the mechanisms underlining the R-loop resolution as well.

In summary, this study shows that during transcription, translocating RNAP by itself can efficiently detect DSBs on DNA templates and initiate the DNA repair process by generating R-loops with the transcripts around the DSB sites and expanding them upstream. The R-loops can become as large as the full-length transcripts involved, thereby completely blocking the next rounds of transcription, in addition to impeding the current round. These findings provide insights into how the cellular TC-HDR process initiates the DNA repair of DSBs on transcription templates in bacteria, as well as possibly archaea and eukaryotes.

## Supplementary Material

gkad689_Supplemental_File

## Data Availability

All data are available from the corresponding authors upon reasonable request.
