# Supplementary material for: Translocating RNA polymerase generates R-loops at DNA double-strand breaks without any additional factors
Source: Nucleic Acids Res. 2023 Aug 28;51(18):9838–48. doi: 10.1093/nar/gkad689 (PMC10570047; doi:10.1093/nar/gkad689)
Supplement: gkad689_Supplemental_File [file gkad689_supplemental_file.pdf]

Supplementary Data

Translocating RNA polymerase generates R-loops at DNA double-strand breaks without any additional factors

Gunhyoung Lim[1], Seungha Hwang[2], Kilwon Yu[2], Jin Young Kang[2], Changwon Kang[3,]* and Sungchul Hohng[1,]*

[1] Department of Physics and Astronomy, and Institute of Applied Physics, Seoul National University, Seoul 08826, Republic of Korea

[2] Department of Chemistry, Korea Advanced Institute of Science and Technology, Daejeon 34141, Republic of Korea

[3] Department of Biological Sciences, and KAIST Stem Cell Center, Korea Advanced Institute of Science and Technology, Daejeon 34141, Republic of Korea

* To whom correspondence should be addressed. Email: shohng@snu.ac.kr
Correspondence may also be addressed to Changwon Kang. Email: ckang@kaist.ac.kr

Contents

Figure S1: FRET changes after transcription resumption
Figure S2: Antibody binding to R-loops
Figure S3: Additional representative time traces for Figure 1
Figure S4: Gradual increase of high-FRET fraction after antibody binding
Figure S5: R-loop degradation by RNase H
Figure S6: Effect of antibody on R-loop efficiency
Figure S7: Cy3-RNA runoff time in the absence of R-loop formation
Figure S8: FRET histograms at the time point of antibody binding to R-loops
Figure S9: Additional representative time traces for Figure 3
Figure S10: FRET changes in the template DT3's scheme II
Figure S11: Histograms of time difference between antibody binding and FRET ascending
Figure S12: Extension of RNA-DNA hybrids
Figure S13: Additional representative time traces for Figure 4
Figure S14: Additional representative time traces for Figure 5
Table S1: DNA oligomers for construction of transcription templates
Table S2: Single-molecule assay data in Figure 2A
Table S3: Single-molecule assay data in Figure 2B
Table S4: Information on the number of molecules and replicated experiments analyzed

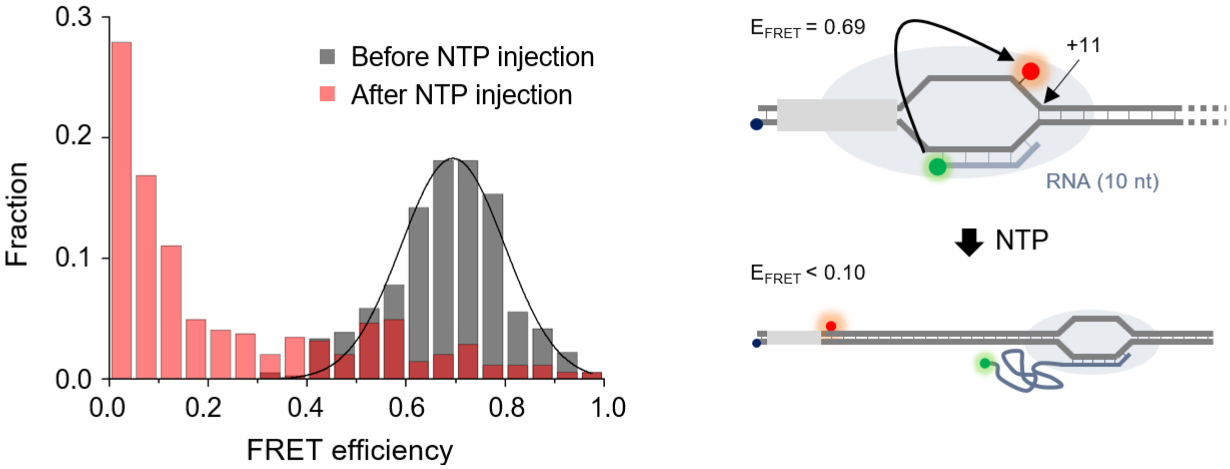

Figure S1. FRET changes after transcription resumption. The elongation complexes of DT1 were examined before NTP injection (gray bars) and 12 s after NTP injection (red bars). A Gaussian function fitted to the gray bars (black line) peaks at $E_{FRET} = 0.69$. This value represents the most frequently observed FRET efficiency before NTP injection.

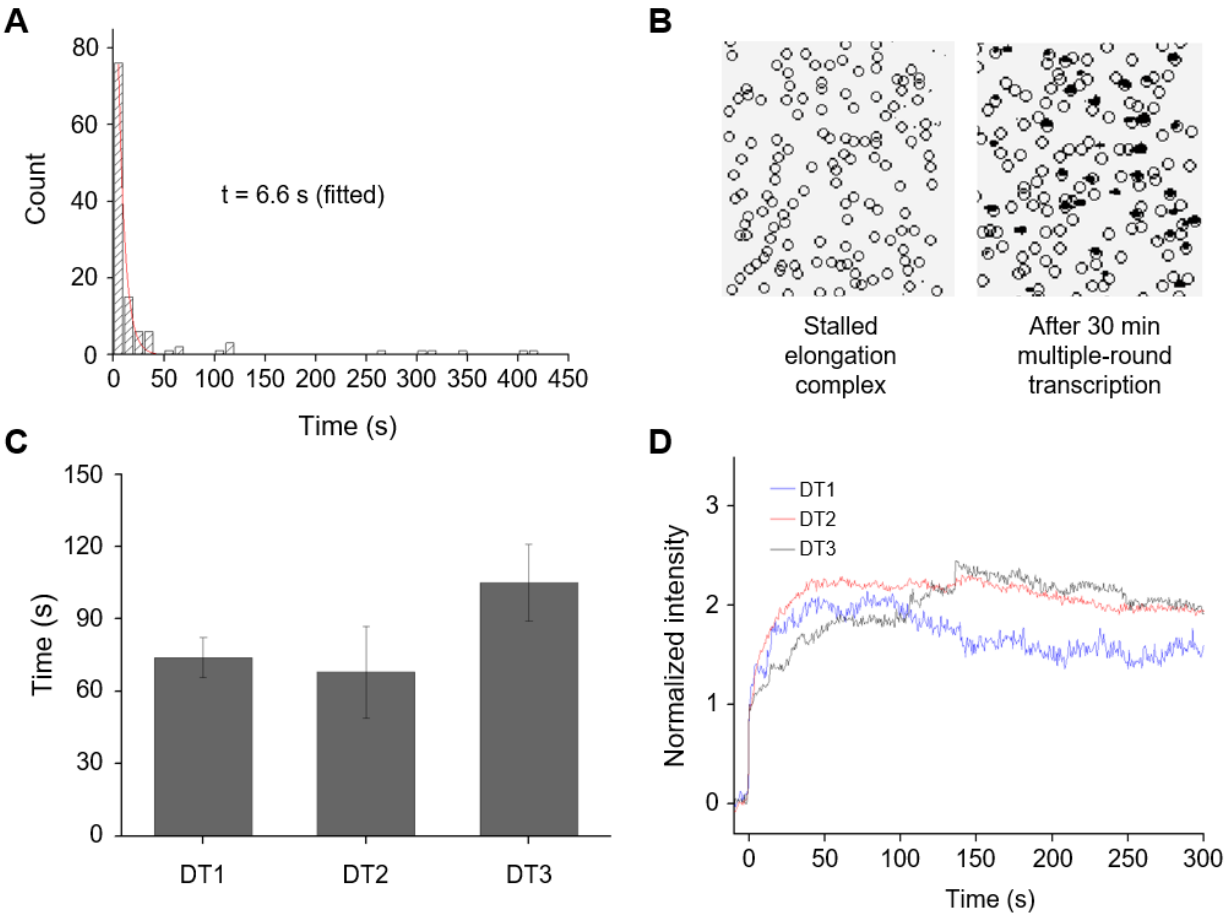

Figure S2. Antibody binding to R-loops. (A) Antibody-R-loop association time. R-loops were generated through 30-min multiple-round transcription of the immobilized stalled elongation complexes, and the time of Alexa-488 signal appearance since the antibody injection was measured. A single-exponential decay curve (red line) revealed a decay time constant of 6.6 s. **(B)** Comparison of Alexa-488 images before (left) and after (right) the antibody binding to R-loops. The antibody was injected to the immobilized stalled elongation complexes of DT3 (left) or injected to the products after 30-min multiple-round transcription (right). The DNA templates are visualized using the Cy5 signal (open circles), and some of them exhibit binding to the antibody (black dots). **(C)** The average time delays from NTP injection to the first antibody binding on DT1, DT2 and DT3. **(D)** Average time traces of Alexa-488 fluorescence of the high-FRET complexes. For the display, the first stable antibody binding was post-synchronized at time zero with fluorescence signals normalized to one. As expected from the sizes of S9.6 antibody and fully-extended R-loop, two or three copies of the antibody complex could bind R-loops at maximum.

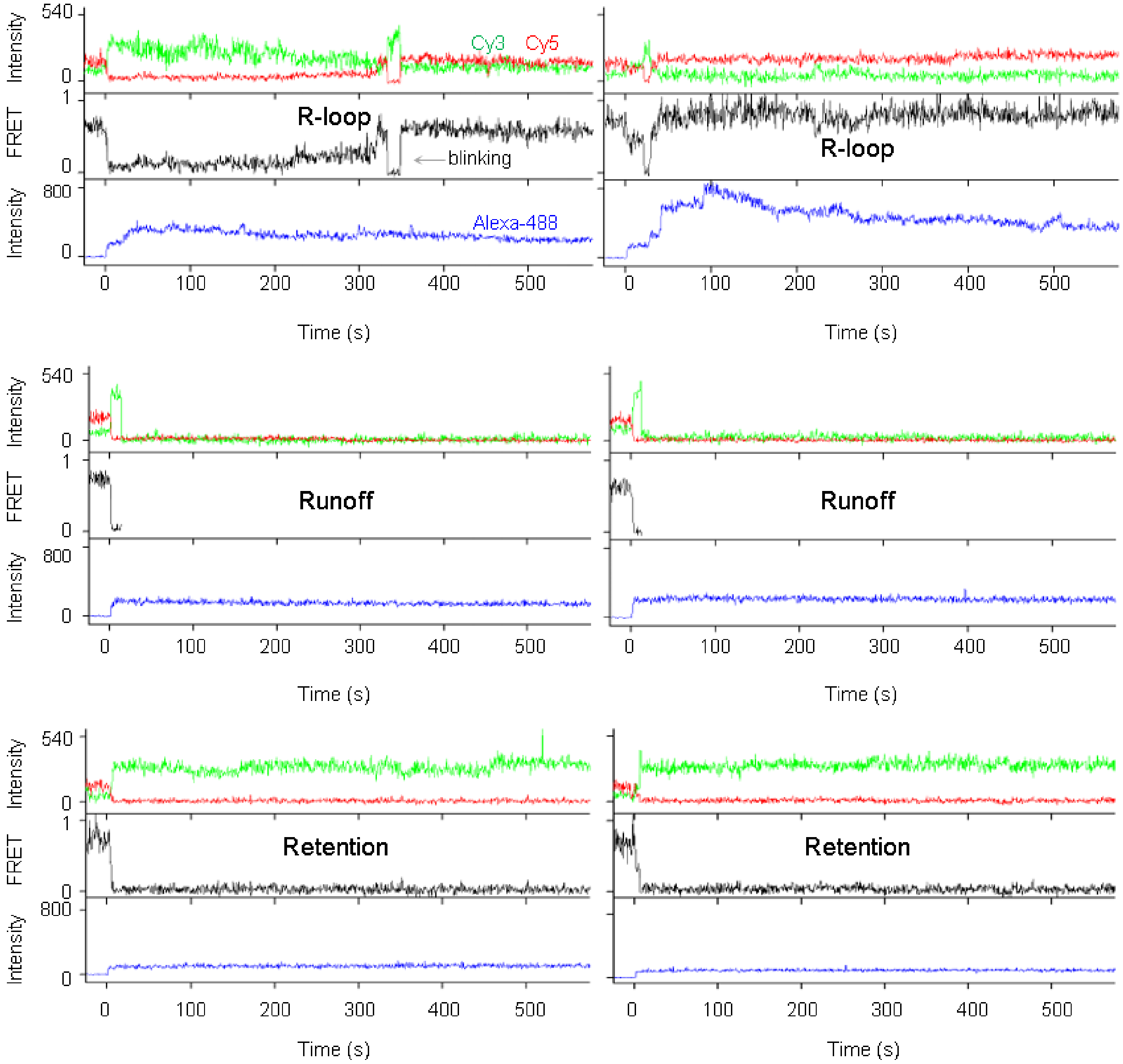

Figure S3. Additional representative time traces for Figure 1. Additional time traces for R-loop, runoff and retention events are presented. Each time trace displays Cy3 and Cy5 fluorescence at Cy3 excitation (top), Cy3-Cy5 FRET (middle), and Alexa-488 fluorescence at its excitation (bottom).

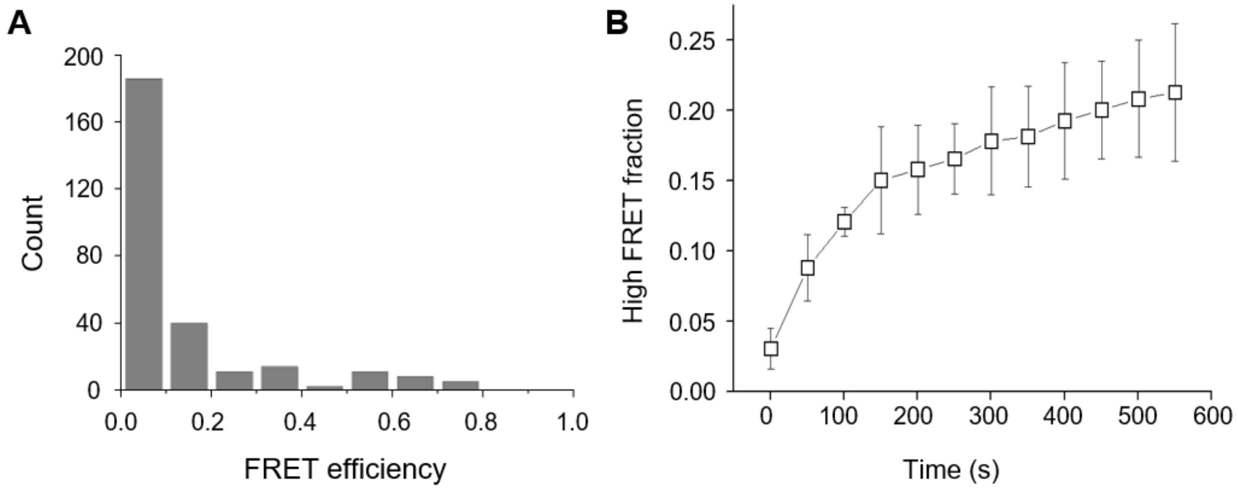

Figure S4. Gradual increase of the high-FRET fraction after antibody binding. (A) The Cy3-Cy5 FRET efficiencies were measured at the time point of the first antibody binding to DT1. **(B)** A high-FRET fraction increases over time since the antibody binding at 0 s. The fraction with E_{FRET} > 0.7 on the y-axis is plotted against the antibody binding time on the x-axis. The error bars represent the standard deviations of three independent experiments.

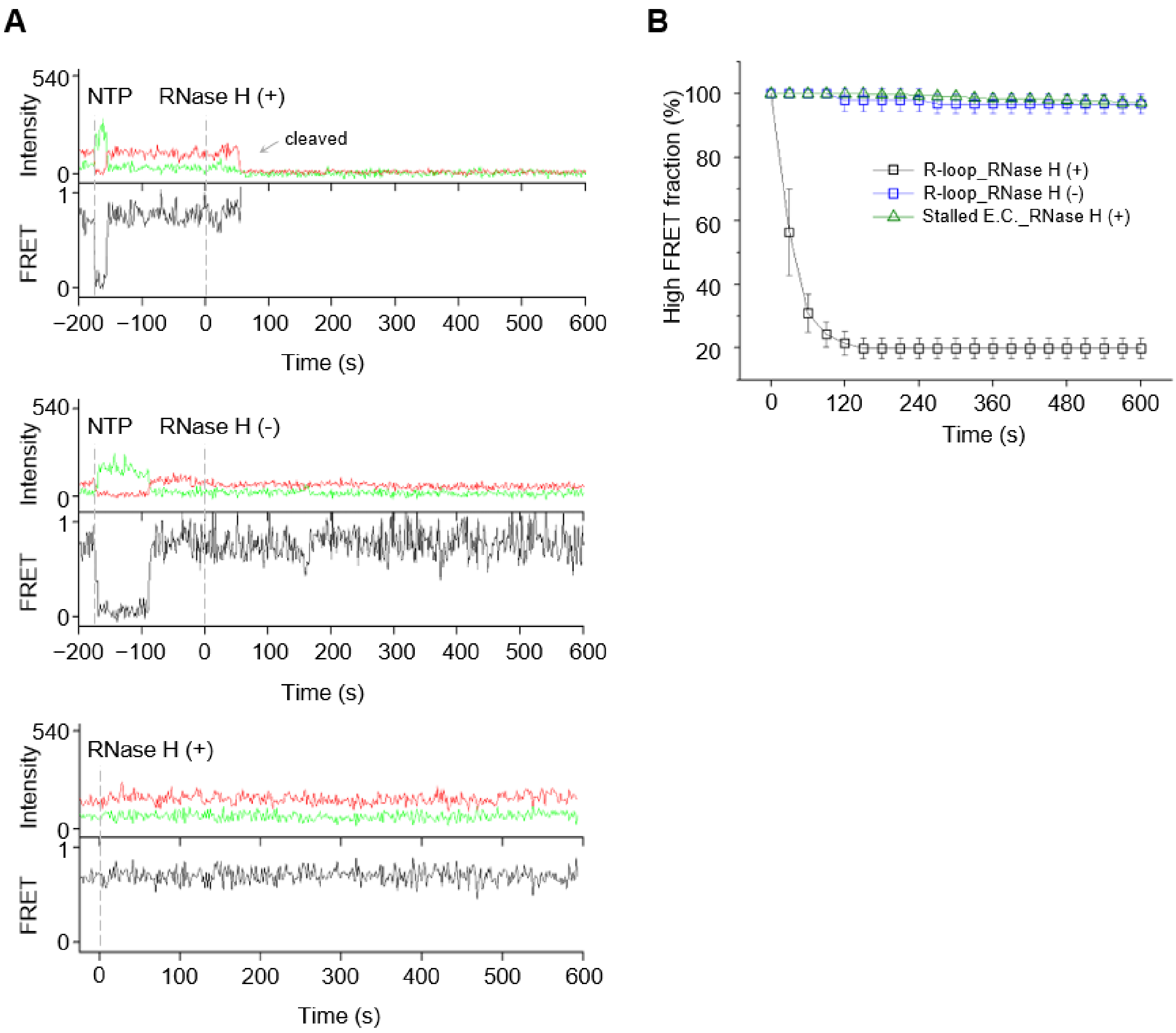

Figure S5. R-loop degradation by RNase H. (A) Shown are the representative time traces obtained from the experiments in which RNase H was added some time after NTP injection (top), from the control experiments where RNase H was not added (middle)or from the experiment of RNase H injection on stalled elongation complex (bottom). In the traces, Cy3 and Cy5 fluorescence at Cy3 excitation and the corresponding FRET are colored in green, red and black, respectively. **(B)** The change of a high FRET population over time. The fraction with E_{FRET} > 0.7 on the y-axis is plotted against the time since the RNase H injection on the x-axis. The error bars represent the standard deviations of three independent experiments.

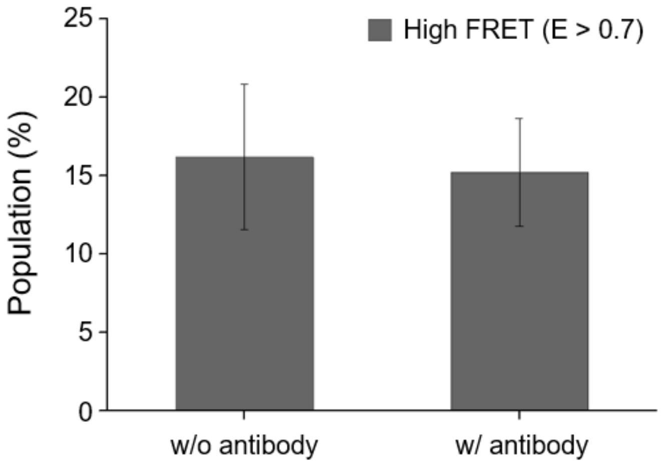

Figure S6. Effect of antibody on R-loop efficiency. The high FRET population with $E_{FRET} > 0.7$ was negligibly affected by the presence of antibody.

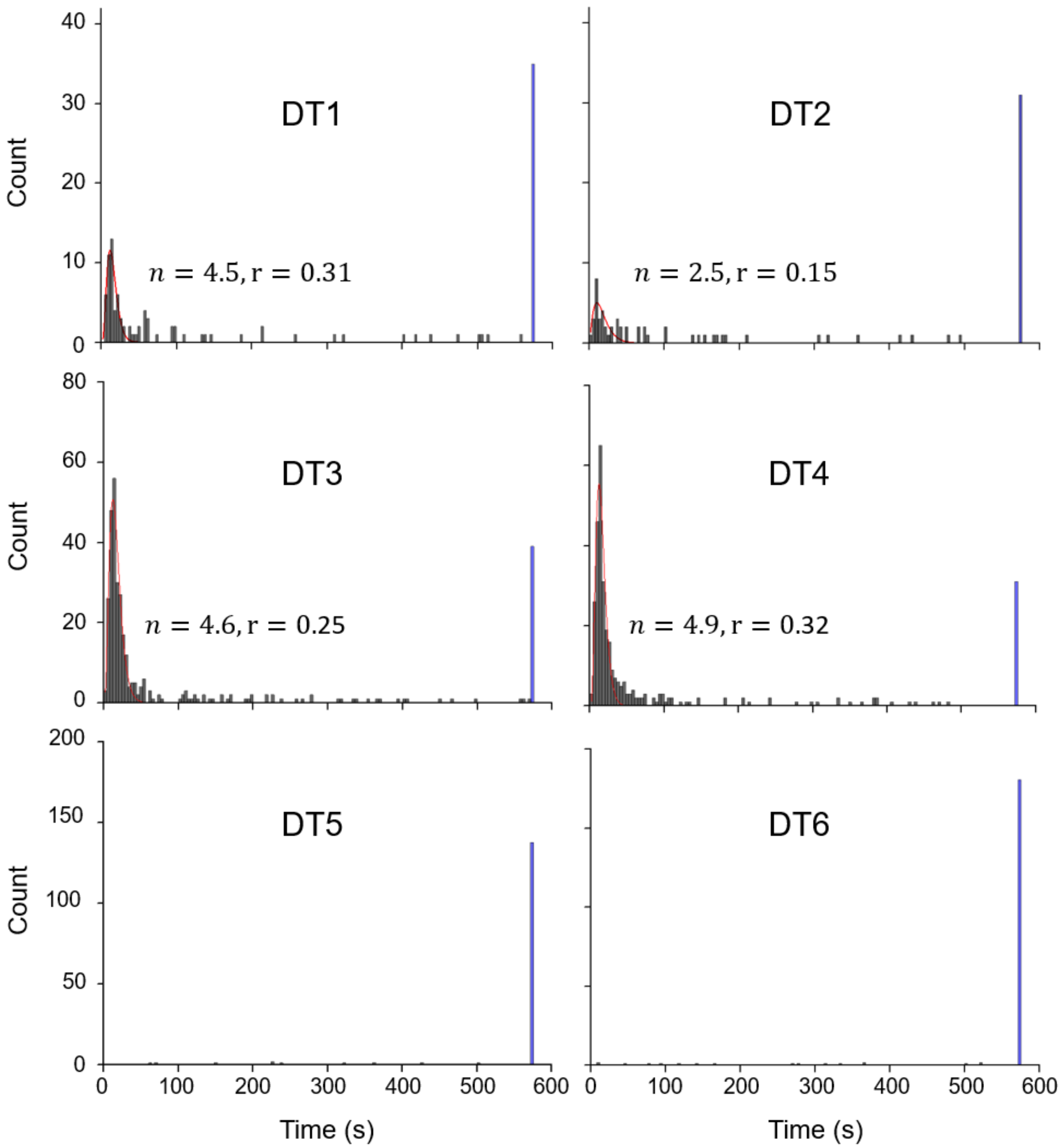

Figure S7. Cy3-RNA runoff time in the absence of R-loop formation. The Cy3-RNA retention duration was measured in the complexes that did not bind the R-loop-specific antibody during a 10-min period. Some complexes retained Cy3-RNA beyond 575 s, and they are represented by a single blue bar at the 575-s timepoint. The remaining complexes, which showed RNA runoff from templates DT1, DT2, DT3 and DT4, were analyzed. The data points are fitted to a gamma distribution (red line) using the equation $y(x) = \frac{A_1 r^n}{\Gamma(n)} x^{n-1} e^{-rx}$. The runoff times (*n*/*r*) were then calculated to be 15 s, 17 s, 15 s and 16 s for templates DT1, DT2, DT3 and DT4, respectively.

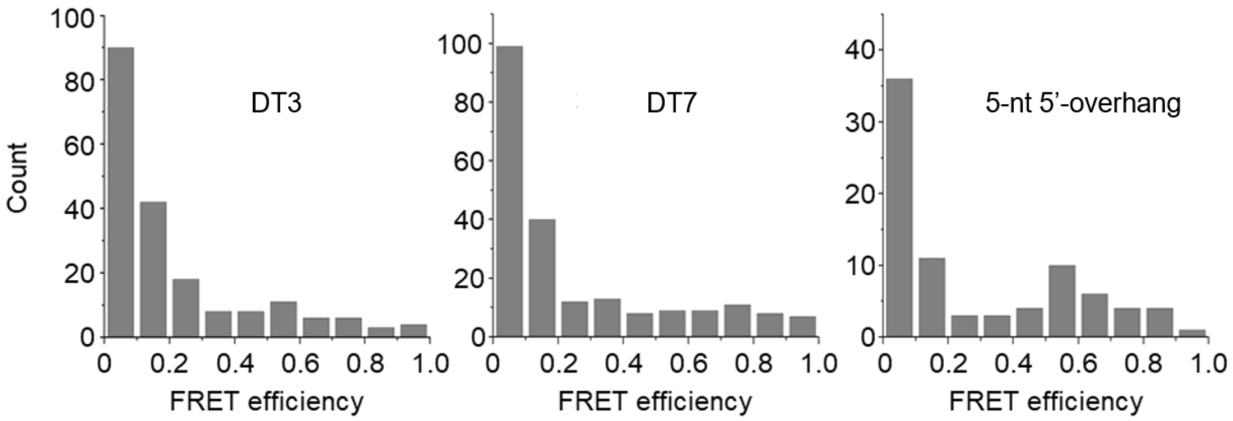

Figure S8. FRET histograms at the time point of the first antibody binding to R-loops. The Cy3-Cy5 FRET efficiencies were measured at the time point of the initial antibody binding to R-loops formed on template DT3 with a blunt end (left), template DT7 with a 5-nt 3’-overhang (center) and the template with a 5-nt 5’-overhang end (right).

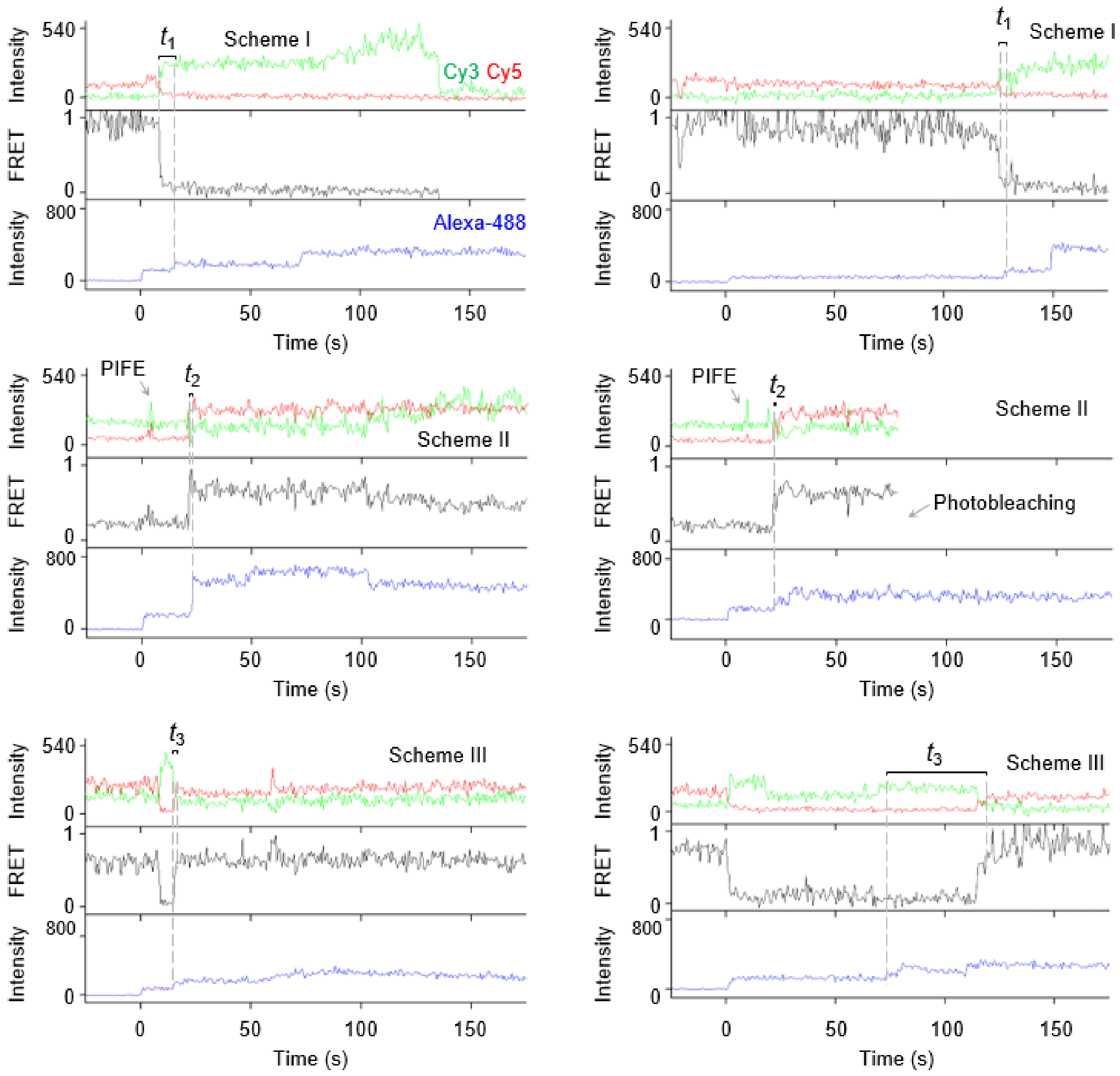

Figure S9. Additional representative time traces for Figure 3. Each time trace displays Cy3 and Cy5 fluorescence at Cy3 excitation (top), Cy3-Cy5 FRET (middle), and Alexa-488 fluorescence at its excitation (bottom). In scheme II, a transient PIFE occurs due to the brief encounter of transcribing RNAP with fluorophores.

Figure S10. FRET changes in the template DT3's scheme II. The Cy3-Cy5 FRET efficiencies were measured before the transcription resumption by NTP injection (gray bars) and after the R-loop formation (red bars) in the template DT3's dye-labeling scheme II. The Gaussian functions (red lines) peak at E_{FRET} = 0.21 and 0.64, respectively.

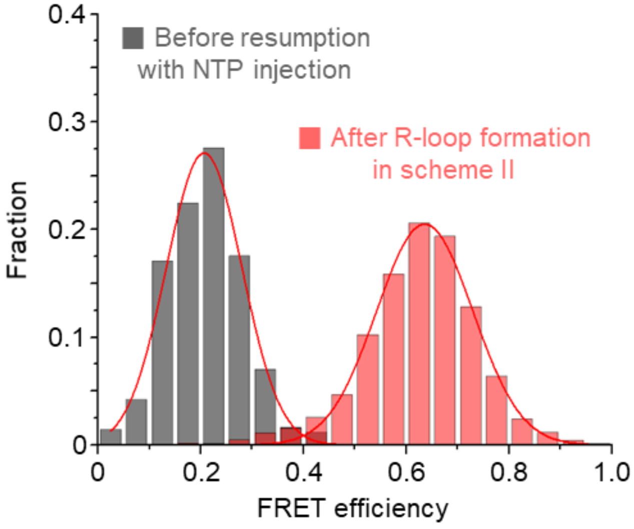

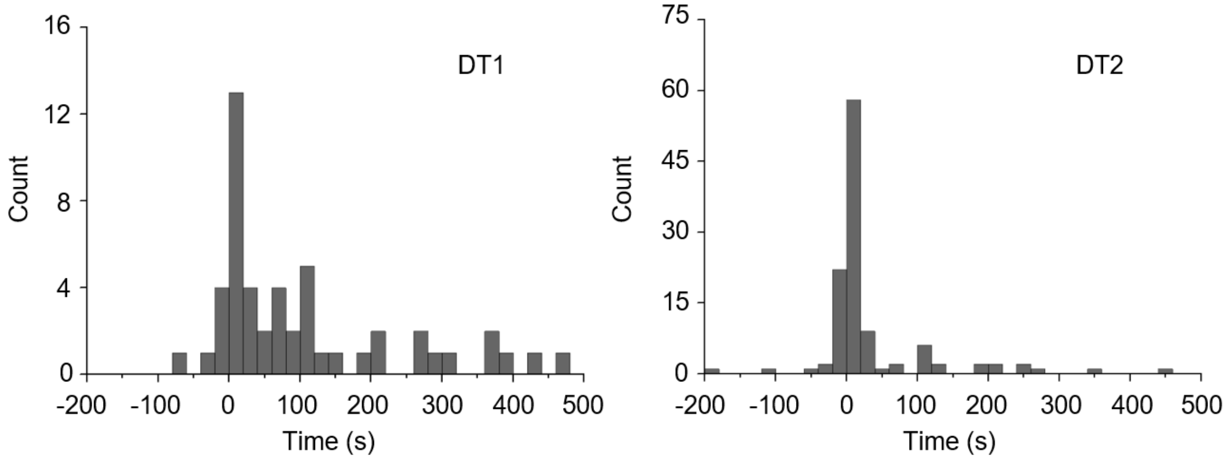

Figure S11. Histograms of time difference between antibody binding and FRET ascending. The average times when E_{FRET} rises above 0.7 after antibody binding are 105 s and 31 s for DT1 and DT2, respectively.

Figure S12. Extension of RNA-DNA hybrids. Using the template DT7 with a 5-nt 3'-overhang end, the FRET changes were timed relative to the antibody detection of R-loops in the three different dye-labeling schemes: scheme I (top), scheme II (middle) and scheme III (bottom), as described Fig. 4A.

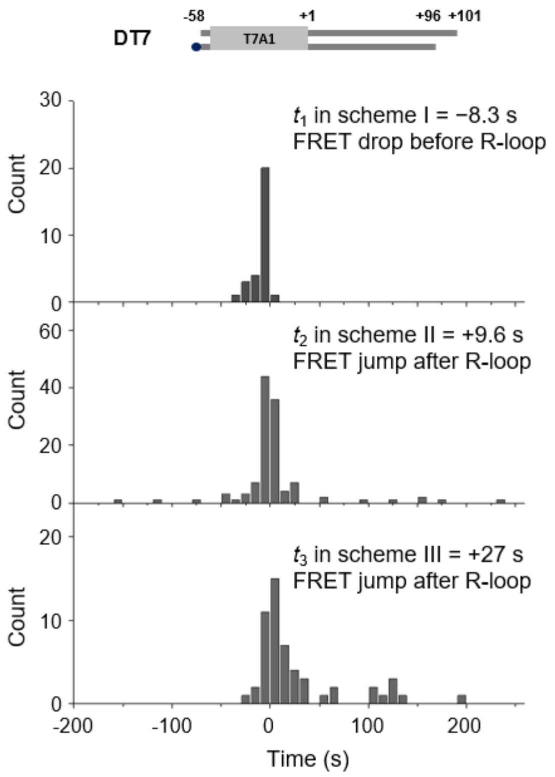

Figure S13. Additional representative time traces for Figure 4. The time trace displays Cy3 and Cy5 fluorescence at Cy3 excitation (top), Cy3-Cy5 FRET (bottom).

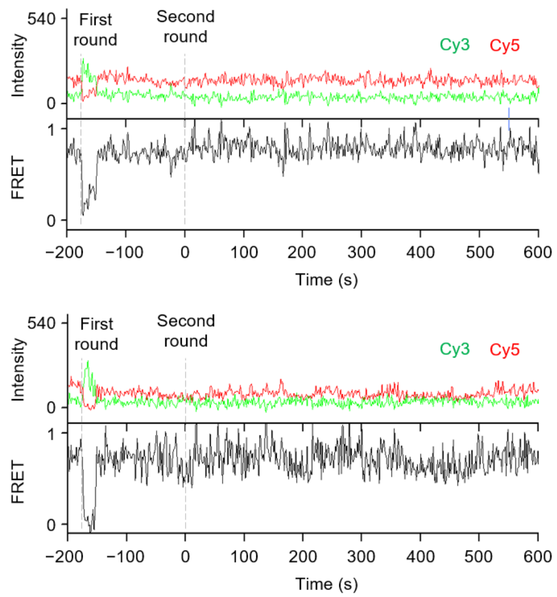

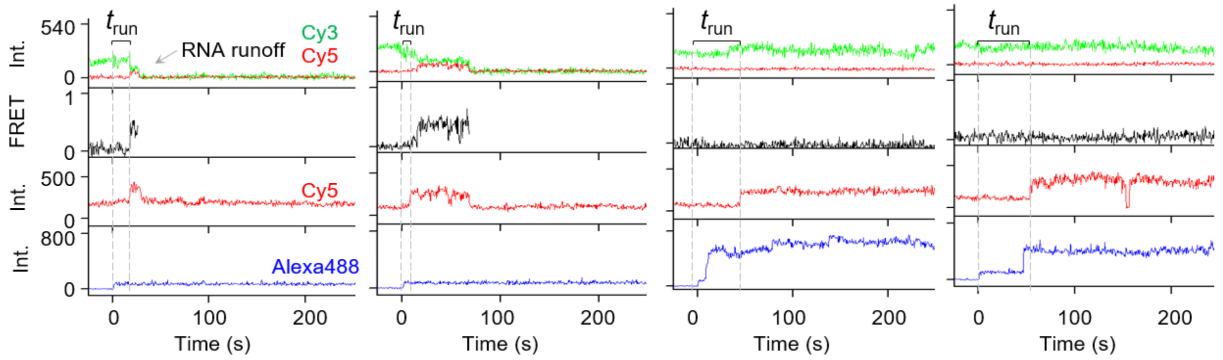

Figure S14. Additional representative time traces for Figure 5. The time trace displays Cy3 and Cy5 fluorescence at Cy3 excitation (1st row), Cy3-Cy5 FRET (2nd row), Cy5 fluorescence at Cy5 excitation (3rd row) and Alexa-488 fluorescence at its excitation (4th row).

Table S1. DNA oligomers for construction of transcription templates

Template	Strand (length)	Sequence (5' to 3')
DT1	Nontemplate (159 nt)	ATCAGGTCTATCAAAAAGAGTATTGACTTAAAGTCTAACCTATAGGATACTTACAGCCATCCT<br>TCTCCAAGGGAGGGA + pGGGAGGGTGTACTGATGCGTTCCACTCGCACCGTCGGACTACCT<br>AGATGCCTATCGTGAGGCGTGCACACTACGTGATGTG
	Splint	CACATCACGTAGTGTGCACGCCTCACGATAGGCATCTAGGTAGTCCGACGGTGCGAGTGGAA<br>CGCATCAGTACACCCTCCCTCCCTCCCTTGGAGAAGGATGGCT
	Template (139 nt)	CCTCACGATAGGCATCTAGGTAGTCCGACGGTGCGAGTGGAACGCATCAGTACACCCTCCCT<br>CCCTCCCTTGGAGAAGGATGGCT + pGTAAGTATCCTATAGGTT<br>AGACTTTAAGTCAATACTCTTTTTGATAGACCTGAT -biotin
	Splint	ATCAGGTCTATCAAAAAGAGTATTGACTTAAAGTCTAACCTATAGGATACTTACAGCCATCCT<br>TCTCCAAGGGAGGGA
DT2	Nontemplate (139 nt)	ATCAGGTCTATCAAAAAGAGTATTGACTTAAAGTCTAACCTATAGGATACTTACAGCCATCCT<br>TCTCCAAGGGAGGGA + pGGGAGGGTGTACTGATGCGTTCCACTCGCACCGTCGGACTACCT<br>AGATGCCTATCGTGAGG
	Splint	CCTCACGATAGGCATCTAGGTAGTCCGACGGTGCGAGTGGAACGCATCAGTACACCCTCCCT<br>CCCTCCCTTGGAGAAGGATGGCT
	Template (159 nt)	CACATCACGTAGTGTGCACGCCTCACGATAGGCATCTAGGTAGTCCGACGGTGCGAGTGGAA<br>CGCATCAGTACACCCTCCCTCCCTCCCTTGGAGAAGGATGGCT + pGTAAGTATCCTATAGGTT<br>AGACTTTAAGTCAATACTCTTTTTGATAGACCTGAT -biotin
	Splint	ATCAGGTCTATCAAAAAGAGTATTGACTTAAAGTCTAACCTATAGGATACTTACAGCCATCCT<br>TCTCCAAGGGAGGGA
DT3	Nontemplate (159 nt)	ATCAGGTCTATCAAAAAGAGTATTGACTTAAAGTCTAACCTATAGGATACTTACAGCCATCCT<br>TCTCCAAGGGAGGGA + pGGGAGGGTGTACTGATGCGTTCCACTCGCACCGTCGGACTACCT<br>AGATGCCTATCGTGAGGCGTGCACACTACGTGATGTG
	Splint	CACATCACGTAGTGTGCACGCCTCACGATAGGCATCTAGGTAGTCCGACGGTGCGAGTGGAA<br>CGCATCAGTACACCCTCCCTCCCTCCCTTGGAGAAGGATGGCT
	Template (159 nt)	CACATCACGTAGTGTGCACGCCTCACGATAGGCATCTAGGTAGTCCGACGGTGCGAGTGGAA<br>CGCATCAGTACACCCTCCCTCCCTCCCTTGGAGAAGGATGGCT + pGTAAGTATCCTATAGGTT<br>AGACTTTAAGTCAATACTCTTTTTGATAGACCTGAT-biotin
	Splint	ATCAGGTCTATCAAAAAGAGTATTGACTTAAAGTCTAACCTATAGGATACTTACAGCCATCCT<br>TCTCCAAGGGAGGGA
DT4	Nontemplate (159 nt)	ATCAGGTCTATCAAAAAGAGTATTGACTTAAAGTCTAACCTATAGGATACTTACAGCCATCCT<br>TCTCCAAATTAATCT + pTAACACATGTACTGATGCGTTCCACTCGCACCGTCGGACTACCTAG<br>ATGCCTATCGTGAGGCGTGCACACTACGTGATGTG
	Splint	CACATCACGTAGTGTGCACGCCTCACGATAGGCATCTAGGTAGTCCGACGGTGCGAGTGGAA<br>CGCATCAGTACATGTGTTAAGATTAATTTGGAGAAGGATGGCT
	Template (159 nt)	CACATCACGTAGTGTGCACGCCTCACGATAGGCATCTAGGTAGTCCGACGGTGCGAGTGGAA<br>CGCATCAGTACATGTGTTAAGATTAATTTGGAGAAGGATGGCT + pGTAAGTATCCTATAGGTT<br>AGACTTTAAGTCAATACTCTTTTTGATAGACCTGAT-biotin
	Splint	ATCAGGTCTATCAAAAAGAGTATTGACTTAAAGTCTAACCTATAGGATACTTACAGCCATCCT<br>TCTCCAAATTAATCT
DT5	Nontemplate (159 nt)	ATCAGGTCTATCAAAAAGAGTATTGACTTAAAGTCTAACCTATAGGATACTTACAGCCATCCT<br>TCTCCAAGGGAGGGA + pGGGAGGGAGAACAGAAGCGAACCACACGCACCGACGGACAAC<br>CAAGAAGCCAAACGAGAGGCGAGCACACAACGAGAAGAGTTTCTTTGTTTCTTT
	Splint	AAAGAAACAAAGAAACTCTTCTCGTTGTGTGCTCGCCTCTCGTTTGGCTTCTTGGTTGTCCG<br>TCGGTGCGTGTGGTTCGCTTCTGTTCTCCCTCCCTCCCTCCCTTGGAGAAGGATGGCT
	Template (159 nt)	AAAGAAACAAAGAAACTCTTCTCGTTGTGTGCTCGCCTCTCGTTTGGCTTCTTGGTTGTCCG<br>TCGGTGCGTGTGGTTCGCTTCTGTTCTCCCTCCCTCCCTCCCTTGGAGAAGGATGGCT +<br>pGTAAGTATCCTATAGGTTAGACTTTAAGTCAATACTCTTTTTGATAGACCTGAT-biotin
	Splint	ATCAGGTCTATCAAAAAGAGTATTGACTTAAAGTCTAACCTATAGGATACTTACAGCCATCCT<br>TCTCCAAGGGAGGGA
DT6	Nontemplate (159 nt)	ATCAGGTCTATCAAAAAGAGTATTGACTTAAAGTCTAACCTATAGGATACTTACAGCCATCCT<br>TCTCCAAGGGAGGGA + pGGGAGGGTGTACTGATGCGTTCCACTCGCACCGTCGG ACTACCT<br>AGATGCCTATCGTGAGGCGTGCACACTACGTGATGTG

	Splint	CACATCACGTAGTGTGCACGCCTCACGATAGGCATCTAGGTAGTCCGACGGTGCGAGTGGAA<br>CGCATCAGTACACCCTCCCTCCCTCCCTTGGAGAAGGATGGCT
	Template<br>(159 nt)	biotin-CACATCACGTAGTGTGCACGCCTCACGATAGGCATCTAGGTAGTCCGACGGTGCGAGT<br>GGA ACGCATCAGTACACCCTCCCTCCCTCCCTTGGAGAAGGATGGCT + pGTAAGTATCCTAT<br>AGGTTAGACTTTAAGTCAATACTCTTTTTGATAGACCTGAT
	Splint	ATCAGGTCTATCAAAAAGAGTATTGACTTAAAGTCTAACCTATAGGATACTTACAGCCATCCT<br>TCTCCAAGGGAGGGA
DT3<br>(scheme I)	Nontemplate<br>(159 nt)	ATCAGGTCTATCAAAAAGAGTATTGACTTAAAGTCTAACCTATAGGATACTTACAGCCATCCT<br>TCTCCAAGGGAGGGA + pGGGAGGGTGTACTGATGCGTTCCACTCGCACCGTCGGACTACCT<br>AGATGCCTATCGTGAGGCGTGCACACTACGTGATGTG-**Cy5**
	Splint	CACATCACGTAGTGTGCACGCCTCACGATAGGCATCTAGGTAGTCCGACGGTGCGAGTGGAA<br>CGCATCAGTACACCCTCCCTCCCTCCCTTGGAGAAGGATGGCT
	Template<br>(159 nt)	**Cy3**-CACATCACGTAGTGTGCACGCCTCACGATAGGCATCTAGGTAGTCCGACGGTGCGAGTG<br>GAACGCATCAGTACACCCTCCCTCCCTCCCTTGGAGAAGGATGGCT + pGTAAGTATCCTATA<br>GGTTAGACTTTAAGTCAATACTCTTTTTGATAGACCTGAT-biotin
	Splint	ATCAGGTCTATCAAAAAGAGTATTGACTTAAAGTCTAACCTATAGGATACTTACAGCCATCCT<br>TCTCCAAGGGAGGGA
DT3<br>(scheme II)	Nontemplate<br>(159 nt)	ATCAGGTCTATCAAAAAGAGTATTGACTTAAAGTCTAACCTATAGGATACTTACAGCCATCCT<br>TCTCCAAGGGAGGGA + pGGGAGGGTGTACTGATGCGTTCCAC + pTCGCACCGTCGGACTA<br>CCTAGATGCCTATCGTGAGGCGTGCACACTACGTGATGTG
	Splint	CACATCACGTAGTGTGCACGCCTCACGATAGGCATCTAGGTAGTCCGACGGTGCGAGTGGAA<br>CGCATCAGTACACCCTCCCTCCCTCCCTTGGAGAAGGATGGCT
	Template<br>(159 nt)	CACATCACGTAGTGTGCACGCCTCACGATAGGCATCTAGGTAGTCCGACGGTGCGAGTGGAA<br>CGCATCAGTACACCCTCCCTCCCTCCCTTGGAGAAGGATGGCT + pGTAAGTATCCTATAGGT<br>TAGACTTTAAGTCAATACTCTTTTTGATAGACCTGAT-biotin
	Splint	ATCAGGTCTATCAAAAAGAGTATTGACTTAAAGTCTAACCTATAGGATACTTACAGCCATCCT<br>TCTCCAAGGGAGGGA
3'-overhang<br>(1 nt)	Nontemplate<br>(159 nt)	ATCAGGTCTATCAAAAAGAGTATTGACTTAAAGTCTAACCTATAGGATACTTACAGCCATCCT<br>TCTCCAAGGGAGGGA + pGGGAGGGTGTACTGATGCGTTCCACTCGCACCGTCGGACTACCT<br>AGATGCCTATCGTGAGGCGTGCACACTACGTGATGTG
	Splint	CACATCACGTAGTGTGCACGCCTCACGATAGGCATCTAGGTAGTCCGACGGTGCGAGTGGAA<br>CGCATCAGTACACCCTCCCTCCCTCCCTTGGAGAAGGATGGCT
	Template<br>(158 nt)	ACATCACGTAGTGTGCACGCCTCACGATAGGCATCTAGGTAGTCCGACGGTGCGAGTGGAAC<br>GCATCAGTACACCCTCCCTCCCTCCCTTGGAGAAGGATGGCT + pGTAAGTATCCTATAGGTTA<br>GACTTTAAGTCAATACTCTTTTTGATAGACCTGAT-biotin
	Splint	ATCAGGTCTATCAAAAAGAGTATTGACTTAAAGTCTAACCTATAGGATACTTACAGCCATCCT<br>TCTCCAAGGGAGGGA
3'-overhang<br>(2 nt)	Nontemplate<br>(159 nt)	ATCAGGTCTATCAAAAAGAGTATTGACTTAAAGTCTAACCTATAGGATACTTACAGCCATCCT<br>TCTCCAAGGGAGGGA + pGGGAGGGTGTACTGATGCGTTCCACTCGCACCGTCGGACTACCT<br>AGATGCCTATCGTGAGGCGTGCACACTACGTGATGTG
	Splint	CACATCACGTAGTGTGCACGCCTCACGATAGGCATCTAGGTAGTCCGACGGTGCGAGTGGAA<br>CGCATCAGTACACCCTCCCTCCCTCCCTTGGAGAAGGATGGCT
	Template<br>(157 nt)	CATCACGTAGTGTGCACGCCTCACGATAGGCATCTAGGTAGTCCGACGGTGCGAGTGGAACG<br>CATCAGTACACCCTCCCTCCCTCCCTTGGAGAAGGATGGCT + pGTAAGTATCCTATAGGTTAG<br>ACTTTAAGTCAATACTCTTTTTGATAGACCTGAT-biotin
	Splint	ATCAGGTCTATCAAAAAGAGTATTGACTTAAAGTCTAACCTATAGGATACTTACAGCCATCCT<br>TCTCCAAGGGAGGGA
3'-overhang<br>(3 nt)	Nontemplate<br>(159 nt)	ATCAGGTCTATCAAAAAGAGTATTGACTTAAAGTCTAACCTATAGGATACTTACAGCCATCCT<br>TCTCCAAGGGAGGGA + pGGGAGGGTGTACTGATGCGTTCCACTCGCACCGTCGGACTACCT<br>AGATGCCTATCGTGAGGCGTGCACACTACGTGATGTG
	Splint	CACATCACGTAGTGTGCACGCCTCACGATAGGCATCTAGGTAGTCCGACGGTGCGAGTGGAA<br>CGCATCAGTACACCCTCCCTCCCTCCCTTGGAGAAGGATGGCT
	Template<br>(156 nt)	ATCACGTAGTGTGCACGCCTCACGATAGGCATCTAGGTAGTCCGACGGTGCGAGTGGAACGC<br>ATCAGTACACCCTCCCTCCCTCCCTTGGAGAAGGATGGCT + pGTAAGTATCCTATAGGTTAGA<br>CTTTAAGTCAATACTCTTTTTGATAGACCTGAT-biotin
	Splint	ATCAGGTCTATCAAAAAGAGTATTGACTTAAAGTCTAACCTATAGGATACTTACAGCCATCCT<br>TCTCCAAGGGAGGGA

3'-overhang (4 nt)	Nontemplate (159 nt)	ATCAGGTCTATCAAAAAGAGTATTGACTTAAAGTCTAACCTATAGGATACTTACAGCCATCCTTCTCCAAGGGAGGGA + pGGGAGGGTGTACTGATGCGTTCCACTCGCACCGTCGGACTACCTAGATGCCTATCGTGAGGCGTGCACACTACGTGATGTG
	Splint	CACATCACGTAGTGTGCACGCCTCACGATAGGCATCTAGGTAGTCCGACGGTGCGAGTGGAACGCATCAGTACACCCTCCCTCCCTCCCTTGGAGAAGGATGGCT
	Template (155 nt)	TCACGTAGTGTGCACGCCTCACGATAGGCATCTAGGTAGTCCGACGGTGCGAGTGGAACGCATCAGTACACCCTCCCTCCCTCCCTTGGAGAAGGATGGCT + pGTAAGTATCCTATAGGTTAGACTTTAAGTCAATACTCTTTTTGATAGACCTGAT-biotin
	Splint	ATCAGGTCTATCAAAAAGAGTATTGACTTAAAGTCTAACCTATAGGATACTTACAGCCATCCTTCTCCAAGGGAGGGA
3'-overhang (5 nt) named DT7	Nontemplate (159 nt)	ATCAGGTCTATCAAAAAGAGTATTGACTTAAAGTCTAACCTATAGGATACTTACAGCCATCCTTCTCCAAGGGAGGGA + pGGGAGGGTGTACTGATGCGTTCCACTCGCACCGTCGGACTACCTAGATGCCTATCGTGAGGCGTGCACACTACGTGATGTG
	Splint	CACATCACGTAGTGTGCACGCCTCACGATAGGCATCTAGGTAGTCCGACGGTGCGAGTGGAACGCATCAGTACACCCTCCCTCCCTCCCTTGGAGAAGGATGGCT
	Template (154 nt)	CACGTAGTGTGCACGCCTCACGATAGGCATCTAGGTAGTCCGACGGTGCGAGTGGAACGCATCAGTACACCCTCCCTCCCTCCCTTGGAGAAGGATGGCT + pGTAAGTATCCTATAGGTTAGACTTTAAGTCAATACTCTTTTTGATAGACCTGAT-biotin
	Splint	ATCAGGTCTATCAAAAAGAGTATTGACTTAAAGTCTAACCTATAGGATACTTACAGCCATCCTTCTCCAAGGGAGGGA
3'-overhang (10 nt)	Nontemplate (159 nt)	ATCAGGTCTATCAAAAAGAGTATTGACTTAAAGTCTAACCTATAGGATACTTACAGCCATCCTTCTCCAAGGGAGGGA + pGGGAGGGTGTACTGATGCGTTCCACTCGCACCGTCGGACTACCTAGATGCCTATCGTGAGGCGTGCACACTACGTGATGTG
	Splint	CACATCACGTAGTGTGCACGCCTCACGATAGGCATCTAGGTAGTCCGACGGTGCGAGTGGAACGCATCAGTACACCCTCCCTCCCTCCCTTGGAGAAGGATGGCT
	Template (149 nt)	AGTGTGCACGCCTCACGATAGGCATCTAGGTAGTCCGACGGTGCGAGTGGAACGCATCAGTACACCCTCCCTCCCTCCCTTGGAGAAGGATGGCT + pGTAAGTATCCTATAGGTTAGACTTTAAGTCAATACTCTTTTTGATAGACCTGAT-biotin
	Splint	ATCAGGTCTATCAAAAAGAGTATTGACTTAAAGTCTAACCTATAGGATACTTACAGCCATCCTTCTCCAAGGGAGGGA
5'-overhang (1 nt)	Nontemplate (158 nt)	ATCAGGTCTATCAAAAAGAGTATTGACTTAAAGTCTAACCTATAGGATACTTACAGCCATCCTTCTCCAAGGGAGGGA + pGGGAGGGTGTACTGATGCGTTCCACTCGCACCGTCGGACTACCTAGATGCCTATCGTGAGGCGTGCACACTACGTGATGT
	Splint	ACATCACGTAGTGTGCACGCCTCACGATAGGCATCTAGGTAGTCCGACGGTGCGAGTGGAACGCATCAGTACACCCTCCCTCCCTCCCTTGGAGAAGGATGGCT
	Template (159 nt)	CACATCACGTAGTGTGCACGCCTCACGATAGGCATCTAGGTAGTCCGACGGTGCGAGTGGAACGCATCAGTACACCCTCCCTCCCTCCCTTGGAGAAGGATGGCT + pGTAAGTATCCTATAGGTTAGACTTTAAGTCAATACTCTTTTTGATAGACCTGAT-biotin
	Splint	ATCAGGTCTATCAAAAAGAGTATTGACTTAAAGTCTAACCTATAGGATACTTACAGCCATCCTTCTCCAAGGGAGGGA
5'-overhang (2 nt)	Nontemplate (157 nt)	ATCAGGTCTATCAAAAAGAGTATTGACTTAAAGTCTAACCTATAGGATACTTACAGCCATCCTTCTCCAAGGGAGGGA + pGGGAGGGTGTACTGATGCGTTCCACTCGCACCGTCGGACTACCTAGATGCCTATCGTGAGGCGTGCACACTACGTGATG
	Splint	CATCACGTAGTGTGCACGCCTCACGATAGGCATCTAGGTAGTCCGACGGTGCGAGTGGAACGCATCAGTACACCCTCCCTCCCTCCCTTGGAGAAGGATGGCT
	Template (159 nt)	CACATCACGTAGTGTGCACGCCTCACGATAGGCATCTAGGTAGTCCGACGGTGCGAGTGGAACGCATCAGTACACCCTCCCTCCCTCCCTTGGAGAAGGATGGCT + pGTAAGTATCCTATAGGTTAGACTTTAAGTCAATACTCTTTTTGATAGACCTGAT-biotin
	Splint	ATCAGGTCTATCAAAAAGAGTATTGACTTAAAGTCTAACCTATAGGATACTTACAGCCATCCTTCTCCAAGGGAGGGA
5'-overhang (3 nt)	Nontemplate (156 nt)	ATCAGGTCTATCAAAAAGAGTATTGACTTAAAGTCTAACCTATAGGATACTTACAGCCATCCTTCTCCAAGGGAGGGA + pGGGAGGGTGTACTGATGCGTTCCACTCGCACCGTCGGACTACCTAGATGCCTATCGTGAGGCGTGCACACTACGTGAT
	Splint	ATCACGTAGTGTGCACGCCTCACGATAGGCATCTAGGTAGTCCGACGGTGCGAGTGGAACGCATCAGTACACCCTCCCTCCCTCCCTTGGAGAAGGATGGCT
	Template (159 nt)	CACATCACGTAGTGTGCACGCCTCACGATAGGCATCTAGGTAGTCCGACGGTGCGAGTGGAACGCATCAGTACACCCTCCCTCCCTCCCTTGGAGAAGGATGGCT + pGTAAGTATCCTATAGGTT

		AGACTTTAAGTCAATACTCTTTTGATAGACCTGAT-biotin
	Splint	ATCAGGTCTATCAAAAAGAGTATTGACTTAAAGTCTAACCTATAGGATACTTACAGCCATCCT<br>TCTCCAAGGGAGGGA
5’-overhang<br>(4 nt)	Nontemplate<br>(155 nt)	ATCAGGTC<u>TATCAAAAAGAGTATTGACTTAAAGTCTAACCTATAGGATACTTACAGCC</u>ATCCT<br>TCTCCAAGGGAGGGA + pGGGAGGGTGTACTGATGCGTTCCACTCGCACCGTCGGACTACCT<br>AGATGCCTATCGTGAGGCGTGCACACTACGTGA
	Splint	TCACGTAGTGTGCACGCCTCACGATAGGCATCTAGGTAGTCCGACGGTGCGAGTGGAACGCA<br>TCAGTACACCCTCCCTCCCTCCCTTGGAGAAGGATGGCT
	Template<br>(159 nt)	CACATCACGTAGTGTGCACGCCTCACGATAGGCATCTAGGTAGTCCGACGGTGCGAGTGGAA<br>CGCATCAGTACACCCTCCCTCCCTCCCTTGGAGAAGGATGGCT + pGTAAGTATCCTATAGGTT<br>AGACTTTAAGTCAATACTCTTTTGATAGACCTGAT-biotin
	Splint	ATCAGGTCTATCAAAAAGAGTATTGACTTAAAGTCTAACCTATAGGATACTTACAGCCATCCT<br>TCTCCAAGGGAGGGA
5’-overhang<br>(5 nt)	Nontemplate<br>(154 nt)	ATCAGGTC<u>TATCAAAAAGAGTATTGACTTAAAGTCTAACCTATAGGATACTTACAGCC</u>ATCCT<br>TCTCCAAGGGAGGGA + pGGGAGGGTGTACTGATGCGTTCCACTCGCACCGTCGGACTACCT<br>AGATGCCTATCGTGAGGCGTGCACACTACGTG
	Splint	CACGTAGTGTGCACGCCTCACGATAGGCATCTAGGTAGTCCGACGGTGCGAGTGGAACGCAT<br>CAGTACACCCTCCCTCCCTCCCTTGGAGAAGGATGGCT
	Template<br>(159 nt)	CACATCACGTAGTGTGCACGCCTCACGATAGGCATCTAGGTAGTCCGACGGTGCGAGTGGAA<br>CGCATCAGTACACCCTCCCTCCCTCCCTTGGAGAAGGATGGCT + pGTAAGTATCCTATAGGTT<br>AGACTTTAAGTCAATACTCTTTTGATAGACCTGAT-biotin
	Splint	ATCAGGTCTATCAAAAAGAGTATTGACTTAAAGTCTAACCTATAGGATACTTACAGCCATCCT<br>TCTCCAAGGGAGGGA
5’-overhang<br>(10 nt)	Nontemplate<br>(149 nt)	ATCAGGTC<u>TATCAAAAAGAGTATTGACTTAAAGTCTAACCTATAGGATACTTACAGCC</u>ATCCT<br>TCTCCAAGGGAGGGA + pGGGAGGGTGTACTGATGCGTTCCACTCGCACCGTCGGACTACCT<br>AGATGCCTATCGTGAGGCGTGCACACT
	Splint	AGTGTGCACGCCTCACGATAGGCATCTAGGTAGTCCGACGGTGCGAGTGGAACGCATCAGTA<br>CACCCTCCCTCCCTCCCTTGGAGAAGGATGGCT
	Template<br>(159 nt)	CACATCACGTAGTGTGCACGCCTCACGATAGGCATCTAGGTAGTCCGACGGTGCGAGTGGAA<br>CGCATCAGTACACCCTCCCTCCCTCCCTTGGAGAAGGATGGCT + pGTAAGTATCCTATAGGTT<br>AGACTTTAAGTCAATACTCTTTTGATAGACCTGAT-biotin
	Splint	ATCAGGTCTATCAAAAAGAGTATTGACTTAAAGTCTAACCTATAGGATACTTACAGCCATCCT<br>TCTCCAAGGGAGGGA
DT8	Primer_F.1_λ<br>(33 nt)	ATACACGAGGTGGTCTGCGGCTCTACCATATCT
	Primer_R.1_λ,<br>total (32 nt)	Cy5-ATACACCCTGTGCTACCAGTTTTAGACGCTCT
	Primer_F.2<br>_Total (71 nt)	ATCAGGTCTATCAAAAAGAGTATTGACTTAAAGTCTAACCTATAGGATACTTACAGCCATCCT<br>TCTCCAAG
	Nontemplate<br>(162 nt)	ATCAGGTCTATCAAAAAGAGTATTGACTTAAAGTCTAACCTATAGGATACTTACAGCCATCCT<br>TCTCCAAGGGAGGGA + pGGGAGGGTGTACTGATGCGTTCCACTCGCACCGTCGGACTACCT<br>AGATGCCTATCGTGAGGCGTGCACACTACGTGATGTGGAG
	Splint	CACATCACGTAGTGTGCACGCCTCACGATAGGCATCTAGGTAGTCCGACGGTGCGAGTGGAA<br>CGCATCAGTACACCCTCCCTCCCTCCCTTGGAGAAGGATGGCT
	Template<br>(159 nt)	CACATCACGTAGTGTGCACGCCTCACGATAGGCATCTAGGTAGTCCGACGGTGCGAGTGGAA<br>CGCATCAGTACACCCTCCCTCCCTCCCTTGGAGAAGGATGGCT + pGTAAGTATCCTATAGGTT<br>AGACTTTAAGTCAATACTCTTTTGATAGACCTGAT
	Splint	ATCAGGTCTATCAAAAAGAGTATTGACTTAAAGTCTAACCTATAGGATACTTACAGCCATCCT<br>TCTCCAAGGGAGGGA

The template and nontemplate strands were each constructed by ligation of two or three pertinent oligonucleotides using a splint DNA.
The T7A1 promoter sequence is underlined in the nontemplate strands.
The red-colored T denotes Cy5-labeled thymine.
The green-colored **T** in the nontemplate strand oligomer for DT3 in scheme II denotes Cy3-labeled thymine.

Table S2. Single-molecule assay data in Figure 2A

Template's downstream end	Overhang length (nt)	R-loop efficiency (%)	*n* in replicated experiments
3'-overhang	1	7.4 ± 0.8	809=213+215+110+271
3'-overhang	2	12 ± 0.7	534=117+257+160
3'-overhang	3	19 ± 0.5	601=237+146+218
3'-overhang	4	32 ± 1.1	846=117+161+164+266+138
3'-overhang (DT7)	5	65 ± 1.7	423=117+138+168
3'-overhang	10	69 ± 1.9	697=77+112+127+208+173
3'-overhang (DT1)	20	73 ± 1.2	484=133+153+198
5'-overhang	1	7.2 ± 0.7	539=159+173+207
5'-overhang	2	8.0 ± 1.0	946=161+222+284+279
5'-overhang	3	7.8 ± 1.0	799=139+231+170+259
5'-overhang	4	7.8 ± 1.0	842=155+159+247+281
5'-overhang	5	13 ± 0.8	649=132+120+181+216
5'-overhang	10	53 ± 1.7	505=182+183+140
5'-overhang (DT2)	20	74 ± 0.8	381=83+119+179

Table S3. Single-molecule assay data in Figure 2B

DNA template	R-loop (%)	Runoff (%)	Retention (%)	*n* in replicated experiments
DT1	73.3 ± 1.2	18.6 ± 0.2	8.1 ± 1.2	484=133+153+198
DT2	73.9 ± 0.8	17.9 ± 0.4	8.2 ± 0.8	381=83+119+179
DT3	8.4 ± 0.9	83.3 ± 2.0	8.3 ± 1.4	699=173+181+97+70+178
DT4	8.9 ± 0.6	83.3 ± 0.5	7.8 ± 0.8	965=178+257+178+169+183
DT5	3.5 ± 0.3	28.4 ± 2.5	68.1 ± 2.5	587=169+225+193
DT6	2.8 ± 0.4	25.0 ± 3.2	72.2 ±3.3	776=229+178+234+135

Table S4. Information on the number of molecules and replicated experiments analyzed

Figure	# of molecules	times of replicated experiments
Figure 3B (top)	35	15
Figure 3B (middle)	52	6
Figure 3B (bottom)	55	13
Figure 4C (RNAP+NTP)	58	6
Figure 4C (RNAP)	58	6
Figure 5B	81	3
Figure 5C	24	9
Figure S1 (before NTP injection)	359	3
Figure S1 (after NTP injection)	344	3
Figure S2A	116	3
Figure S2C (DT1)	336	3
Figure S2C (DT2)	306	3
Figure S2C (DT3)	44	5
Figure S2D (DT1)	41	3
Figure S2D (DT2)	99	3
Figure S2D (DT3)	52	13
Figure S4A	278	3
Figure S4B	251	3
Figure S5B (RNase H +)	67	3
Figure S5B (RNase H -)	51	3
Figure S5B (Stalled E.C.)	375	3
Figure S6 (w/o antibody)	377	3
Figure S6 (w/ antibody)	365	3
Figure S7 (DT1)	116	3
Figure S7 (DT2)	84	3
Figure S7 (DT3)	340	5
Figure S7 (DT4)	332	5
Figure S7 (DT5)	293	3
Figure S7 (DT6)	358	4
Figure S8 (DT3)	195	14
Figure S8 (DT7)	216	3
Figure S8 (5 nt 5’-overhang)	82	5
Figure S10 (before NTP injection)	57	6
Figure S10 (after R-loop forms)	31	6
Figure S11(DT1)	50	3
Figure S11(DT2)	114	3
Figure S12 (top)	29	3
Figure S12 (middle)	116	4
Figure S12 (bottom)	54	3
